# Silphiperfolene-Type Terpenoids and Other Metabolites from Cultures of the Tropical Ascomycete *Hypoxylon rickii* (Xylariaceae)

**DOI:** 10.1007/s13659-015-0065-3

**Published:** 2015-06-16

**Authors:** Frank Surup, Eric Kuhnert, Elena Liscinskij, Marc Stadler

**Affiliations:** Department Microbial Drugs, Helmholtz Centre for Infection Research GmbH, Inhoffenstraße 7, 38124 Braunschweig, Germany; German Centre for Infection Research (DZIF), Partner Site Hannover-Braunschweig, 38124 Braunschweig, Germany

**Keywords:** Hypoxylon, Xylariaceae, Natural products, Secondary metabolites, Structure elucidation

## Abstract

**Abstract:**

A culture isolated from ascospores of *Hypoxylon rickii*, a xylariaceous ascomycete collected in Martinique, had yielded botryane, noreremophilane and abietane-type terpenoids in a preceding study, but additional metabolites were detected by extensive HPLC–MS analysis in other fractions. Herein we report the further isolation of four new sesquiterpenoids with a silphiperfol-6-ene skeleton from extracts of *H. rickii*. The planar structures were elucidated by NMR and HRMS data as 13-hydroxysilphiperfol-6-ene (**1**), 9-hydroxysilphiperfol-6-en-13-oic acid (**2**), 2-hydroxysilphiperfol-6-en-13-oic acid (**3**) and 15-hydroxysilphiperfol-6-en-13-oic acid (**4**). For compounds **2**–**4** we propose the trivial names rickinic acids A–C. Their stereochemistry was assigned by ROESY correlations as well as by the specific optical rotation. Additionally, the known compounds, botryenanol, dehydrobotrydienol, cyclo(Phe-Pro), cyclo(Pro-Leu), (+)-ramulosin and α-eleostearic acid were isolated. The antimicrobial and cytotoxic activities of the new compounds are also reported.

**Graphical Abstract:**

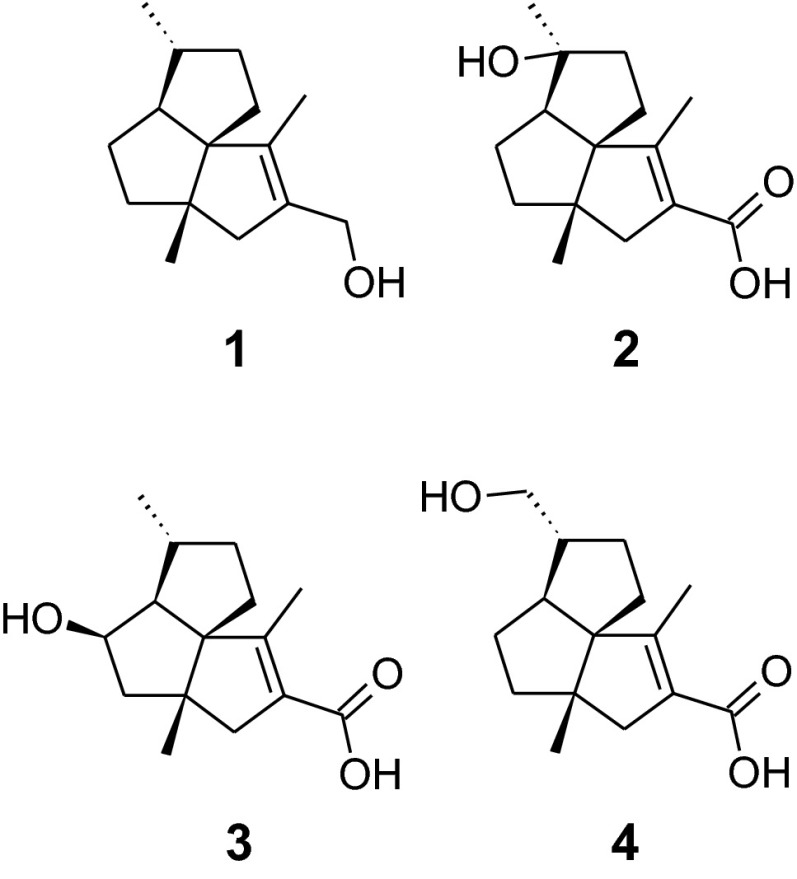

**Electronic supplementary material:**

The online version of this article (doi:10.1007/s13659-015-0065-3) contains supplementary material, which is available to authorized users.

## Introduction

The exploration of the highly diverse fungal family Xylariaceae in terms of secondary metabolite production has revealed a tremendous amount of natural products from most of the biosynthetic pathways. Whereas polyketides and PKS-NRPS hybrid molecules occur in the stromata [1–6] and cultures of the family members [7, 8], terpenes were so far exclusively reported from cultures. Important examples of the latter structural class produced by the Xylariaceae are the antifungal sordarins from *Rosellinia* and *Hypoxylon* spp. [9], the neuropeptide gamma receptor antagonists, xylarenals A and B from *Xylaria* [10], the phytotoxic hymatoxins from “*Hypoxylon*” (current name *Entoleuca*) *mammatum* [11], the antihypertensive vinigrol from *Virgaria* [12], the antibacterial hypocoprins from *Hypocopra* [13], or botryanes from *Daldinia concentrica* [14].

Although the genus *Hypoxylon* is one of the largest within the Ascomycota and its representatives are frequently encountered as endophytes, little is known about their secondary metabolite production capabilities in cultures. Recently, we evaluated the diversity of natural products produced by the ex-epitype strain of *H. rickii*. Subsequently, various terpenoids of the botryane, noreudesmane and abietane scaffold were isolated from a single large scale fermentation of the strain [15]. We now report the isolation, structure elucidation and biological activity of four new silphiperfolene-type terpenoids and six known natural products from the same fungus.

## Results and Discussion

**Fig. 1:**
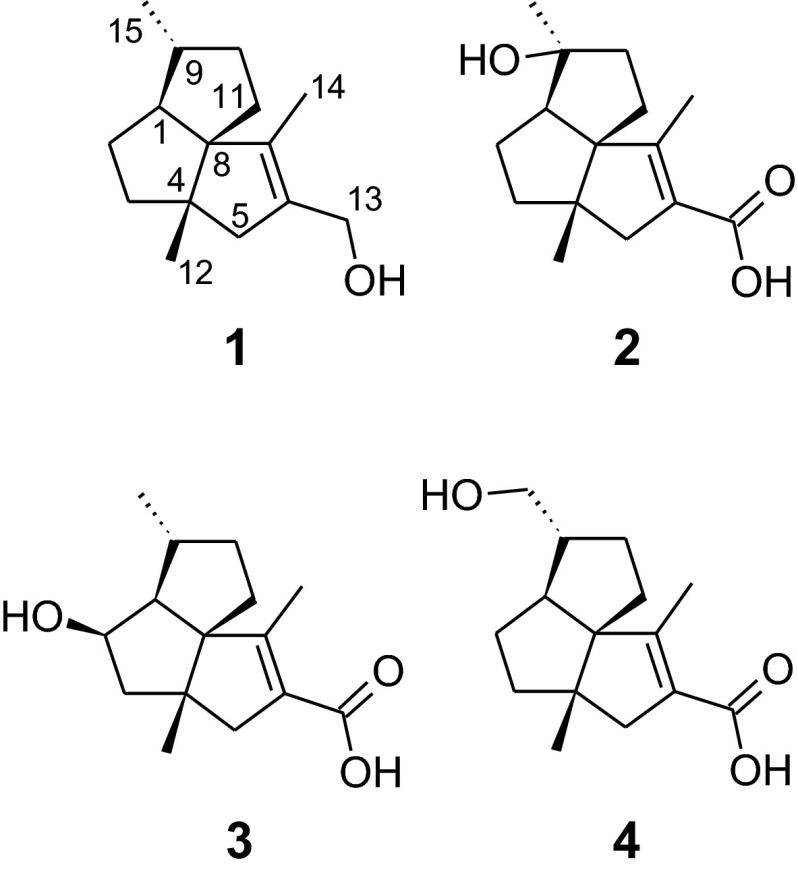
Structures of new metabolites **1**–**4**

**Fig. 2:**
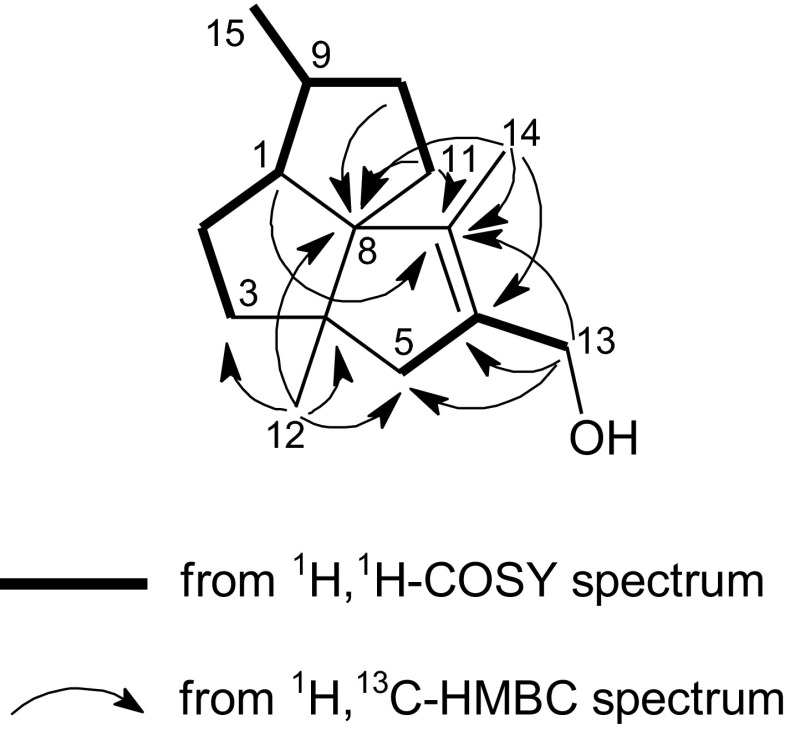
Selected 2D NMR correlations of **1**

A 70 L fermentation of a *H. rickii* strain was processed by separating the mycelia from the culture broth and extraction of the corresponding biomass with acetone. The crude extract was pre-fractionized by MPLC and we focused on the isolation of the hydrophobic components by using HPLC. Besides several not further characterized fatty acids, we isolated the new metabolite **1** (Fig. 1) by subsequent HPLC. Compound **1** was obtained as a colorless oil; its molecular formula C_15_H_24_O was deduced from the molecular ion cluster [M+H]^+^ at *m/z* 219.1737 in the HRESIMS spectrum, which is implying 4 degrees of unsaturation. Proton and ^1^H,^13^C-HSQC NMR experiments revealed the presence of three methyls, six methylenes (one of which was oxygenated) and two methines. Furthermore, the carbon NMR spectrum suggested two *sp*^2^ hybridized and two *sp*^3^ hybridized quaternary carbons. The structural backbone of **1** was determined by ^1^H,^1^H COSY and ^1^H,^13^C HMBC correlations. Starting form methyl H_3_-15 the extensive spin system H_2_-11/H_2_-10/H-9(H_3_-15)/H-1/H_2_-2/H_2_-3 was determined by ^1^H,^1^H COSY and TOCSY correlations (Fig. 2). By ^1^H,^13^C HMBC correlations from methylenes H_2_-5 and H_2_-13 along with methyl H_3_-14 the C-5/C-6(C-13)/C-7/C-14 partial structure was determined. Finally, ^1^H,^13^C HMBC correlations from H-1, H_2_-2, H_2_-3, H_2_-5, H_2_-9, H_2_-10, H_2_-11, H_3_-12, H_3_-14 to quaternary carbon C-8 demonstrated the 13-hydroxysilphiperfol-6-ene structure. ^1^H,^1^H ROESY correlations between H_3_-15 and H_β_-11 on the β-face and H_3_-12 and H_α_-11 on the α-face specify the typical 1S*,4S*,8S*,9R* configuration of silphiperfolene-type sesquiterpenoids. Because compound **1** bears no additional optical centers, the negative specific optical rotation of **1** defines the absolute configuration as 1*S*,4*S*,8*S*,9*R*, since (−)-silphiperfol-6-ene has been synthesized from (*R*)-(+)-pulegone [16].

**Fig. 3:**
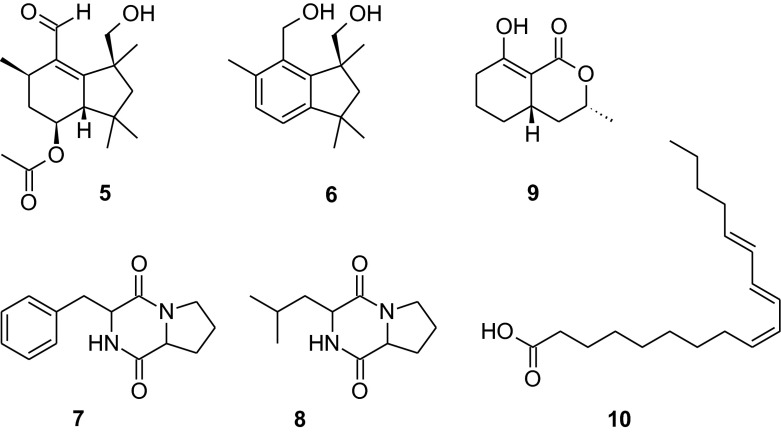
Structures of known metabolites isolated from *H. rickii*

To provide additional material *H. rickii* was cultivated in 10 L scale in YM medium. 13-Hydroxysilphiperfol-6-ene (**1**) could not be detected, but three oxidized derivatives (**2**–**4**) were isolated by preparative MPLC and HPLC. Metabolite **2** was obtained as a colorless oil; its molecular formula C_15_H_22_O_3_ was determined by the [M+H]^+^ molecular ion cluster at *m/z* 251.1649 in the HRESIMS spectrum. The main difference in the proton spectrum of **2** compared to **1** was the shortfall of signals for methine H-9 and methylene H_2_-13. This result was confirmed by the carbon spectrum, in which additional signals for a carboxyl and oxygenated quaternary *sp*^3^ hybridized carbon atoms were observed. ^1^H,^13^C HMBC correlations from H_2_-5 to C-13 identified the carboxylic acid carbon atom as C-13. Singulet methyl H_3_-15 showed ^1^H,^13^C HMBC correlations to oxygen bearing C-9 besides C-1 and C-10. Therefore, **2** was identified as 9-hydroxysilphiperfol-6-en-13-oic acid. The configuration of stereocenter C-9 was assigned as *S* as a consequence of the ^1^H,^1^H ROESY correlation between methyls H_3_-15 and H_3_-14. This correlation demonstrates that the oxygenation occurred with retention of the configuration of methyl group CH_3_-15. **2** was named rickinic acid A.

Rickinic acid B (**3**) has the same molecular formula C_15_H_22_O_3_ as **2**, which was obtained by HRESIMS data. Proton, carbon and ^1^H,^13^C HSQC NMR spectra were highly similar to that of **2**. However, the key differences were the replacement of the methylene signal for C-2 by an oxygenated methine, and the replacement of oxygenated quaternary carbon C-9 by a methine. Therefore, **3** was identified as 2-hydroxysilphiperfol-6-en-13-oic acid. The ^1^H,^1^H ROESY correlations from H-2 to H-1/H_β_-3 and H_β_-5 on the β-face respectively from H_3_-12 to H_α_-3 and H_α_-5 on the α-face of the molecule indicated a 2*R* configuration.

With rickinic acid C (**4**) another metabolite was isolated with a C_15_H_22_O_3_ molecular formula. The proton and ^1^H,^13^C HSQC NMR spectra showed the oxidation of methyl CH_3_-15 to a hydroxymethyl group, thus identifying **4** as 15-hydroxysilphiperfol-6-en-13-oic acid.

Besides the new metabolites **1**–**4**, we isolated several known metabolites (Fig. 3) botryenanol (**5**), dehydrobotrydienol (**6**), cyclo(Phe-Pro) (**7**), cyclo(Pro-Leu) (**8**), (+)-ramulosin (**9**) and α-eleostearic acid (**10**) from different cultivations of *H. rickii* [17–22].

The bioactivity of **1**–**4** was evaluated against *S. cerevisiae*, *C. albicans*, *B. subtilis*, *E. coli* and the mouse fibroblast cell line L929. We detected weak antifungal activity of **2** against the yeast *S. cerevisiae* (MIC = 66.7 µg/mL), weak antibacterial activity of **4** against *B. subtilis* (MIC = 33.3 µg/mL) and weak cytotoxic activity of **3** (IC_50_ = 20 µg/mL).

The sesquiterpenoids **1**–**4** belong to a family of compounds with a silphiperfolene core structure. These compounds are known to be produced by members of the plant family Asteraceae [23, 24]. However, no fungal metabolite with this particular core structure has been described to the best of our knowledge. The role of these metabolites for *H.* *rickii* in nature remains elusive. The compounds might be produced to modulate its host plant, similarly to the fungal production of gibberellin-type phytohormons [25]. Though, the silphiperfolene metabolites might also be side products in the biosynthesis of botryanes. Presilphiperfolanol has been proposed as precursor of triquinane [26] as well as botryane [27] type sesquiterpenes. Even though presilphiperfolanol as potential common precursor has not been detected so far, this might explain the co-occurrence of silphiperfolene and botryane-type metabolites in cultures of *H. rickii*.

The identification of compounds **5** and **6** continues the list of known botryane-type terpenoids *from H. rickii* [15]. This class of secondary metabolites has already been reported from other xylariaceous fungi like *Daldinia concentrica* and a *Geniculisporium* sp. [14, 28] and seems therefore common within the family. The same is true for the isocoumarin derivative (+)-ramulosin (**9**) which frequently occurs in the genus *Hypoxylon* [29]. The latter has been shown to exhibit phytotoxic and antifungal effects [30]. The production of diketopiperazines in the Xylariaceae is so far only known from *Rosellinia necatrix* [31] and therefore **7** and **8** are the first report of this particular compound class from another member of the family.

## Experimental

### General Experimental Procedures

Optical rotations were determined with a Perkin-Elmer 241 spectrometer and UV spectra were recorded with a Shimadzu UV–Vis spectrophotometer UV-2450. NMR spectra were recorded with Bruker Avance III 700 spectrometer with a 5 mm TCI cryoprobe (^1^H 700 MHz, ^13^C 175 MHz) and Avance III 500 (1H 500 MHz, 13C 125 MHz) spectrometers. HRESIMS mass spectra were obtained as previously described [32]. Isolation of pure compounds was achieved if not indicated otherwise with a preparative HPLC (Gilson, Middleton, USA) equipped with a GX-271 Liquid Handler, a 172 DAD, a 305 and 306 pump (with 50SC Piston Pump Head). As stationary phase a VP Nucleodur C18 ec column (125 × 40 mm, 7 µm; Macherey–Nagel) was used. The mobile phase was composed of deionised water (Milli-Q, Millipore, Schwalbach, Germany) with 0.1 % acetic acid (solvent A1; Roth) and acetonitrile (ACN) with 0.1 % acetic acid (solvent B1). Flow rate was set to 15 ml/min.

### Fungal Material

Stromata (fruiting bodies) of *H. rickii* MJF10324 were collected in 2010 from the Caribbean island Martinique by J. Fournier. The strain was designated as epitype of the species [33]. The culture was derived by multispore isolation on YMG medium (1.0 % malt extract, 0.4 % glucose, 0.4 % yeast extract, pH 6.3) using the method outlined by Stadler et al. [34] and has been deposited in public culture collections (MUCL 53309, CBS 129345).

### Cultivation in 70 L Scale and Isolation of **1** and **10**

Large-scale fermentation of the strain was carried out as previously described [14] in HLX media (3.0 % sucrose, 1.0 % casamino acids, 0.1 % K_2_HPO_4_, 0.1 % yeast extract, 0.05 % MgSO_4_ × 7H_2_O, 0.05 % KCl, 0.001 % FeSO_4_ × 7H_2_O). The fermentation was aborted after 7 days as sugars (sucrose, fructose) were depleted. Compound **1** and **10** were obtained from the acetone crude extract of the biomass (12 g) by preparative RP MPLC and subsequent HPLC. The conditions for the RP MPLC were as follows: a ODS/AQ C18 column (480 × 30 mm, Kronlab) as stationary phase, mobile phase composed of solvent A2 [90 % deionised water (Milli-Q), 10 % methanol] and solvent B2 (methanol), linear gradient of solvent B2 from 10 to 100 % in 60 min, followed by isocratic conditions at 100 % solvent B2 for 20 min, flow rate of 30 ml/min, UV peak detection at 210 nm. Compound **1** (2 mg) was purified from the MPLC fraction with a retention time (RT) of 58.5–64.0 min (390 mg) using a linear gradient from 55 to 100 % solvent B1 in 25 min followed by isocratic conditions at 100 % for 15 min at a RT = 26.0 min. Compound **10** (RT: 14.0 min; 2 mg) was isolated from another MPLC fraction (RT: 64.0–67.0 min; 1.8 g) by RP HPLC (linear gradient from 90 to 100 % B1 in 20 min, followed by 15 min isocratic conditions).

13-Hydroxysilphiperfol-6-ene (**1**): amorphous powder, [α]_D_^25^−29 (*c* 0.2 MeOH); ^1^H (700 MHz) and ^13^C (175 MHz) NMR data (methanol-*d*_4_), see Tables 1 and 2; HRESIMS: *m/z* 203.1791 (calcd for C_15_H_23_, [M+H−H_2_0]^+^, 203.1794), 219.1737 (calcd for C_15_H_23_O, [M+H]^+^, 219.1743).

**Table 1:** ^13^C data of **1** (175 MHz, methanol-*d*_4_) and **2**–**4** (175 MHz, chloroform-*d*)

	**1**	**2**	**3**	**4**
1	60.5, CH	60.3, CH	63.1, CH	54.3, CH
2	29.9, CH_2_	25.0, CH_2_	72.5, CH	30.3, CH_2_
3	40.6, CH_2_	41.7, CH_2_	47.3, CH_2_	40.1, CH_2_
4	50.8, C	49.8, C	45.1, C	49.5, C
5	49.2, CH_2_	46.5, CH_2_	47.7, CH_2_	46.4, CH_2_
6	132.4, C	123.3, C	123.4, C	123.1, C
7	141.1, C	162.6, C	162.4, C	162.7, C
8	73.4, C	73.8, C	73.5, C	73.7, C
9	42.8, CH	80.0, C	34.9, CH	49.9, CH
10	37.8, CH_2_	43.2, CH_2_	37.2, CH_2_	31.6, CH_2_
11	30.9, CH_2_	28.3, CH_2_	30.3, CH_2_	30.0, CH_2_
12	24.9, CH_3_	23.1, CH_3_	24.7, CH_3_	23.8, CH_3_
13	59.4, CH_2_	169.0, C	168.4, C	168.0, C
14	11.0, CH_3_	13.4, CH_3_	13.7, CH_3_	13.7, CH_3_
15	19.8, CH_3_	28.7, CH_3_	21.2, CH_3_	66.2, CH_2_

**Table 2:** ^1^H data of **1** (700 MHz, methanol-*d*_4_) and **2**–**4** (700 MHz, chloroform-*d*)

	**1**	**2**	**3**	**4**
1	1.66, m	2.07, m	1.90, m	1.99, td (7.7, 2.6)
2α 2β	1.39, m 1.71, m	1.62, m 1.71, m	4.33, ddd (10.3, 7.7, 6.0)	1.46, m 1.77, m
3α 3β	1.52, m 1.65, m	1.61, m 1.64, m	1.58, dd (12.5, 10.3) 1.92, dd (12.5, 6.0)	1.56, m 1.63, m
5α 5β	2.13, dq (16.0, 1.7) 2.37, dq (16.0, 2.2)	2.36, dd (16.0, 1.7) 2.53, dd (16.0, 2.2)	2.39, dd (16.5, 1.7) 2.52, dd (16.5, 2.2)	2.35, dq (16.2, 1.7) 2.54, dq (16.2, 2.2)
9	1.47, m		2.10, m	1.73, m
10α 10β	1.77, m 1.26, m	1.76, m 1.76, m	1.85, m 1.35, m	1.85, m 1.49, m
11α 11β	1.63, m 1.56, m	1.85, m 1.50, m	1,68, m 1.68, m	1.68, m 1.64, m
12	1.03, s	1.08, s	1.02, s	1.05, s
13	4.04, s			
14	1.61, dd (2.2, 1.7)	2.06, dd (2.2, 1.7)	2.05, dd (2.2, 1.7)	2.03, dd (2.2, 1.7)
15	0.99, d (6.5)	1.32, s	1.06, d (6.5)	3.64, dd (10.5, 5.8) 3.55, dd (10.5, 7.5)

α-Eleostearic acid (**10**): colorless oil; ^13^C NMR (chloroform-*d*, 125 MHz) *δ* 14.1, 22.4, 24.8, 28.0, 29.1, 29.2, 29.7, 29.9, 31.5, 32.7, 33.9, 126.2, 129.0, 130.5, 132.1, 133.2, 135.5, 178.8; HRESIMS: *m/z* 279.2319 (calcd for C_18_H_31_O_2_, [M+H]^+^, 279.2319); spectroscopic and spectrometric data are in good agreement with the literature [22].

### Cultivation in 10 L Scale and Isolation of **2**–**4** and **6**–**8**

A seed culture of the strain with a total volume of 200 mL was prepared in YMG medium (1.0 % malt extract, 0.4 % glucose, 0.4 % yeast extract, pH 6.3), incubated at 22 °C and 140 rpm for 5 days, homogenized with an ultratorax and incubated again for 2 days (22 °C, 140 rpm). A BR 15.4 bioreactor (B. Braun Melsungen AG, Germany) filled with 10 L YMG medium and supplemented with 0.5 % talcum powder (Sigma-Aldrich, St. Louis, USA) was inoculated with 100 mL of the seed culture. The temperature was set at 26 °C. The stirrer speed was set to 150 rpm, aeration rate was set to 0.07 vvm and remained constant during fermentation. The culture was harvested after 7 days as sugars (sucrose, fructose) were depleted. Thereafter, the mycelium was separated from the culture fluid by vacuum filtration to yield a total amount of 525 g wet biomass, which was later extracted with 1.8 L acetone in an ultrasonic bath for 1 h. The acetone extract was filtered and evaporated to yield an aqueous phase, which was further processed by extraction with 3 × 100 mL ethyl acetate in a separating funnel. Subsequently the organic phases were combined and evaporated to yield 221 mg of oily mycelial crude extract (ME) in total.

The culture filtrate was extracted by 2 % XAD-16 (200 g) over 2 h at room temperature. The XAD was separated by filtration and extracted with methanol (500 mL). The extract was evaporated to yield an aqueous phase, which was further processed by extraction with 3 × 50 mL ethyl acetate. The combined organic extracts were filtrated over Strata X column to yield 643 mg crude extract.

The crude extract was subjected to silica gel chromatography using gradient elution with portions of dichloromethane/methanol mixtures (250 mL) of 100/0, 99/1, 98/2, 97/3, 95/5, 90/10, 85/15, 80/20, 70/30, 50/50, 25/75, 0/100 to give 12 fractions. Fraction 2 (303 mg) was fractionized with preparative HPLC (PLC 2020, Gilson, Middleton, USA). A VP Nucleodur C_18_ ec column (150 × 40 mm, 7 µm; Macherey–Nagel) was used as stationary phase. The mobile phase was composed of deionised water (Milli-Q) as solvent A and methanol (MeOH) as solvent B. A flow rate of 30 ml min^−1^ was used for the following gradient: 10–80 % solvent B in 30 min, afterwards in 5 min to 100 % B, isocratic conditions at 100 % for 10 min. UV detection was carried out at 210 and 254 nm and fractions were collected and combined according to the observed peaks. **2** (0.9 mg) was obtained at a retention time (RT) = 30.7–31.0 min.

Fraction 5 (89 mg) was subjected to preparative HPLC as described above (but gradient: 30–100 % solvent B in 30 min, isocratic conditions at 100 % for 10 min) to yield **3** (0.6 mg) at a RT = 18.7–21.7 min and **4** (0.3 mg) at RT = 14.3–15.5 min.

Fraction 1 (242 mg) subjected to preparative HPLC as described above (but gradient: 0–70 % solvent B in 40 min, afterwards in 5 min to 100 % B, isocratic conditions at 100 % for 5 min) to yield **7** (0.8 mg) and **8** at a RT = 19.6–20.2 and RT = 17.7–18.6 min, respectively. Fractions from 35.1 to 36.4 min were combined and fractionated again (gradient: 40–80 % acetonitril in 40 min, afterwards in 5 min to 100 % ACN, isocratic conditions at 100 % for 5 min, VP Nucleodur C_18_ ec column (250 × 21 mm, 7 µm; Macherey–Nagel) to yield **6** (2.4 mg) at a RT = 20.5–22 min.

Rickinic acid A (**2**): amorphous powder, [α]_D_^25^ +2.2 (*c* 0.02 CHCl_3_); UV (MeOH) λ_max_ (log ε) 234 nm (4.7); ^1^H (700 MHz) and ^13^C (175 MHz) NMR data (chloroform-*d*), see Tables 1 and 2; ESIMS: *m/z* 250.95 [M+H]^+^, 248.86 [M−H]^−^; HRESIMS: *m/z* 251.1649 (calcd for C_15_H_23_O_3_,[M+H]^+^, 251.1642).

Rickinic acid B (**3**): amorphous powder, [α]_D_^25^ +12 (*c* 0.1 CHCl_3_); UV (MeOH) λ_max_ (log ε) 234 nm (4.5); ^1^H (700 MHz) and ^13^C (175 MHz) NMR data (chloroform-*d*), see Tables 1 and 2; ESIMS: *m/z* 251.02 [M+H]^+^, 248.96 [M−H]^−^; HRESIMS: *m/z* 251.1648 (calcd for C_15_H_23_O_3_,[M+H]^+^, 251.1642).

Rickinic acid C (**4**): amorphous powder, [α]_D_^25^ +11 (*c* 0.1 CHCl_3_); UV (MeOH) λ_max_ (log ε) 220 nm (sh); ^1^H (700 MHz) and ^13^C (175 MHz) NMR data (chloroform-*d*), see Tables 1 and 2; ESIMS: *m/z* 233.01 [M+H-H_2_0]^+^, 248.94 [M−H]^−^; HRESIMS: *m/z* 251.1648 (calcd for C_15_H_23_O_3_,[M+H]^+^, 251.1642).

Dehydrobotrydienol (**6**): colorless oil; ^13^C NMR (chloroform-*d*, 125 MHz) *δ* 19.0, 26.3, 31.1, 32.2, 40.9, 50.4, 54.0, 58.8, 71.1, 123.1, 130.5, 134.4, 136.4, 144.1, 152.3; HRESIMS: *m/z* 257.1517 (calcd for C_15_H_22_O_2_Na, [M+H]^+^, 257.1512); spectroscopic and spectrometric data are in good agreement with the literature [18].

Cyclo(Phe-Pro) (**7**): colorless oil; ^13^C NMR (chloroform-*d*, 125 MHz) *δ* 22.6, 28.4, 36.8, 45.5, 56.2, 59.2, 127.6, 129.1, 129.4, 135.9, 165.1, 169.5; HRESIMS: *m/z* 245.1297 (calcd for C_14_H_17_N_2_O_2_, [M+H]^+^, 245.1285); spectroscopic and spectrometric data are in good agreement with the literature [19].

Cyclo(Pro-Leu) (**8**): colorless oil; ^13^C NMR (chloroform-*d*, 125 MHz) *δ* 21.2, 22.8, 23.3, 24.8, 28.2, 29.7, 38.7, 45.6, 53.4, 59.0, 166.1, 170.3; HRESIMS: *m/z* 211.1461 (calcd for C_11_H_19_N_2_O_2_, [M+H]^+^, 211.1441); spectroscopic and spectrometric data are in good agreement with the literature [20].

### Small-Scale Cultivation and Isolation of **5** and **9**

Two submerged cultures were grown in each case in 200 mL YMG medium supplemented with 3 g talcum powder on a rotary shaker at 140 rpm and 23 °C. Cultures were harvested after 12 days as free glucose was consumed. Afterwards the supernatant was separated from the biomass by vacuum filtration and extracted with 200 mL ethyl acetate. The organic phases were combined, dried over sodium sulfate and evaporated to yield 50 mg crude extract. The latter was fractionated by RP HPLC using the following conditions: a VP 250/21 Nucleodur100-5 C18 ec column (Macherey–Nagel) equipped with a Kromasil 100 C18 pre-column (50 × 20 mm, 7 μm; AkzoNobel) as stationary phase, solvents A1 and B1 as mobile phase, linear gradient from 20 to 70 % solvent B1 in 40 min, then from 70 to 100 % B1 in 5 min, followed by 10 min isocratic conditions, flow rate of 15 ml/min. Compound **5** (1.4 mg) was obtained at a RT = 33.0 min and **9** (1.5 mg) at a RT = 35.0 min.

Botryenanol (**5**): Colorless oil; ^13^C NMR (chloroform-*d*, 125 MHz) *δ* 20.9, 21.4, 23.8, 29.1, 29.3, 36.8, 39.1, 51.8, 54.2, 58.5, 70.5, 71.9, 139.0, 164.7, 170.3, 192.6; HRESIMS: *m/z* 317.1728 (calcd for C_17_H_26_O_4_Na, [M+H]^+^, 317.1723); spectroscopic and spectrometric data are in good agreement with the literature [17].

(+)-Ramulosin (**9**): Colorless amorphous powder, [α]_D_^25^ +12 (*c* 0.1 CH_3_OH); ^13^C NMR (chloroform-*d*, 125 MHz) *δ* 21.0, 21.8, 29.1, 29.6, 33.0, 37.5, 76.6, 96.8, 171.9, 174.8; HRESIMS: *m/z* 183.1026 (calcd for C_10_H_15_O_3_, [M+H]^+^, 183.1016); spectroscopic and spectrometric data are in good agreement with the literature [21].

### Biological Assays

Antibacterial, antifungal and cytotoxic assays were performed as described by Surup et al. [35].

## Electronic supplementary material

Supplementary material 1 (PDF 1293 kb)
