# Supplementary material for: Silphiperfolene-Type Terpenoids and Other Metabolites from Cultures of the Tropical Ascomycete Hypoxylon rickii (Xylariaceae)
Source: Nat Prod Bioprospect. 2015 Jun 16;5(3):167–73. doi: 10.1007/s13659-015-0065-3 (PMC4488154; doi:10.1007/s13659-015-0065-3)
Supplement: Supplementary file 1 — Supplementary material 1 (PDF 1293 kb) [file 13659_2015_65_MOESM1_ESM.pdf]

Silphiperfolene-type terpenoids and other metabolites from cultures of the tropical ascomycete *Hypoxylon rickii* (Xylariaceae)

Frank Surup[a,b] , Eric Kuhnert[a,b] , Elena Liscinskij[a] and Marc Stadler[a,b,] *

a Helmholtz Centre for Infection Research GmbH, Department Microbial Drugs, Inhoffenstraße 7, 38124 Braunschweig, Germany.

b German Centre for Infection Research (DZIF), partner site Hannover-Braunschweig, 38124 Braunschweig, Germany.

* Corresponding author. Tel.: +49 531 6181-4240; fax: +49 531 6181 9499; e-mail: marc.stadler@helmholtz-hzi.de

--- Supporting Information ---

Table of Contents

HPLC-HRESIMS data of **1.** S4

^{1}H NMR spectrum (500 MHz, CH_3OH-d_4) of **1**. S5

^{13}C NMR spectrum (125 MHz, CH_3OH-d_4) of **1**. S6

COSY NMR spectrum (500 MHz, CH_3OH-d_4) of **1**. S7

ROESY NMR spectrum (500 MHz, CH_3OH-d_4) of **1**. S8

HSQC NMR spectrum (500 MHz, CH_3OH-d_4) of **1**. S9

HMBC NMR spectrum (500 MHz, CH_3OH-d_4) of **1**. S10

HPLC-HRESIMS data of **2**. S11

^{1}H NMR spectrum (700 MHz, $CHCl_3$-d) of **2**. S12

^{13}C NMR spectrum (175 MHz, $CHCl_3$-d) of **2**. S13

COSY NMR spectrum (700 MHz, $CHCl_3$-d) of **2**. S14

ROESY NMR spectrum (700 MHz, $CHCl_3$-d) of **2**. S15

HSQC NMR spectrum (700 MHz, $CHCl_3$-d) of **2**. S16

HMBC NMR spectrum (700 MHz, $CHCl_3$-d) of **2**. S17

HPLC-HRESIMS data of **3**. S18

^{1}H NMR spectrum (700 MHz, $CHCl_3$-d) of **3**. S19

^{13}C NMR spectrum (175 MHz, $CHCl_3$-d) of **3**. S20

COSY NMR spectrum (700 MHz, $CHCl_3$-d) of **3**. S21

ROESY NMR spectrum (700 MHz, $CHCl_3$-d) of **3**. S22

HSQC NMR spectrum (700 MHz, $CHCl_3$-*d*) of **3**. S23
HMBC NMR spectrum (700 MHz, $CHCl_3$-*d*) of **3**. S24
HPLC-HRESIMS data of **4**. S25
^{1}H NMR spectrum (700 MHz, $CHCl_3$-*d*) of **4**. S26
^{13}C NMR spectrum (175 MHz, $CHCl_3$-*d*) of **4**. S27
COSY NMR spectrum (700 MHz, $CHCl_3$-*d*) of **4**. S28
ROESY NMR spectrum (700 MHz, $CHCl_3$-*d*) of **4**. S29
HSQC NMR spectrum (700 MHz, $CHCl_3$-*d*) of **4**. S30
HMBC NMR spectrum (700 MHz, $CHCl_3$-*d*) of **4**. S31

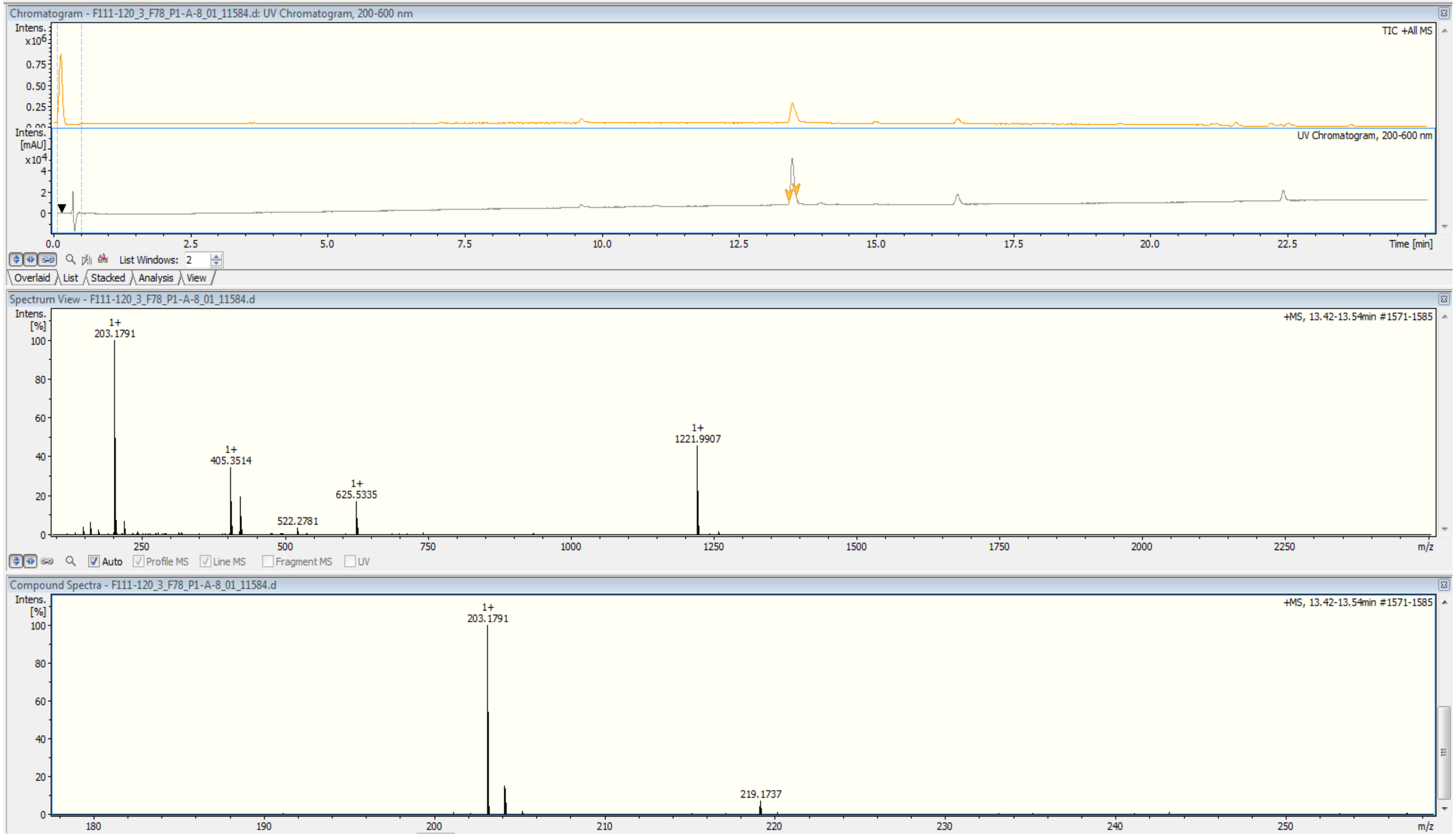

HPLC-HRESIMS data of **1**.

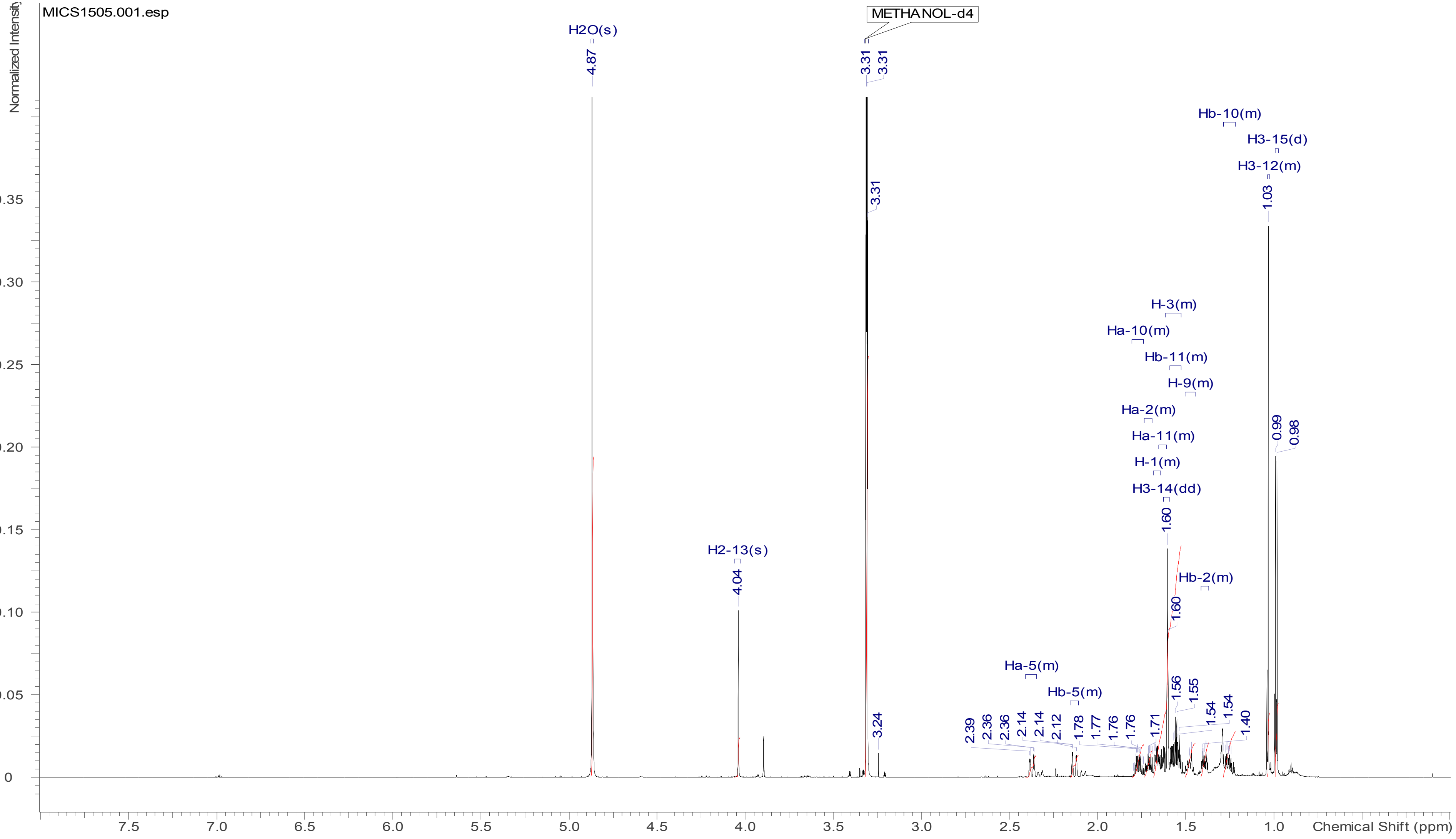

^{1}H NMR spectrum (500 MHz, CH_3OH-d_4) of **1**.

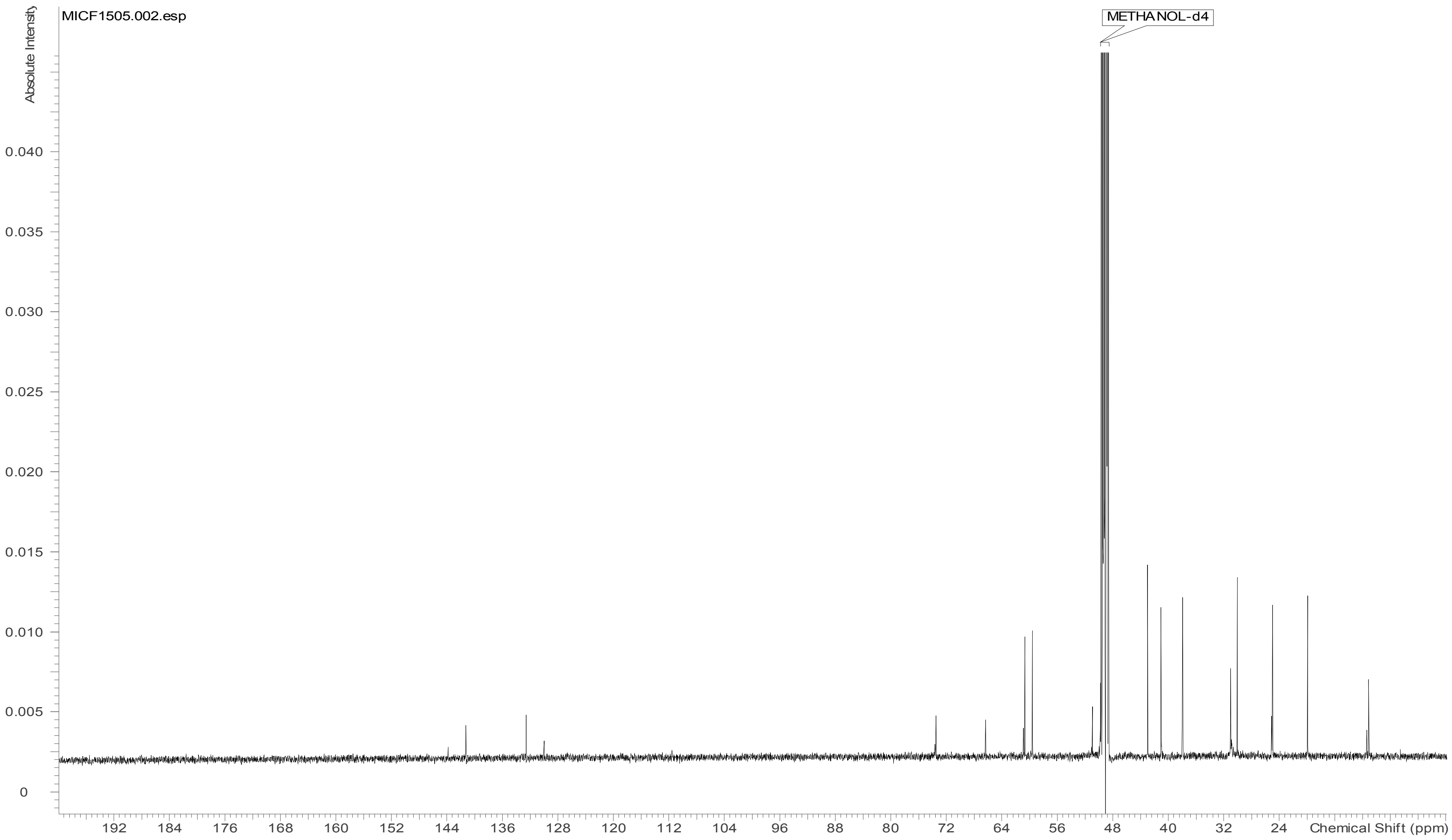

^{13}C NMR spectrum (125 MHz, CH_3OH-d_4) of **1**.

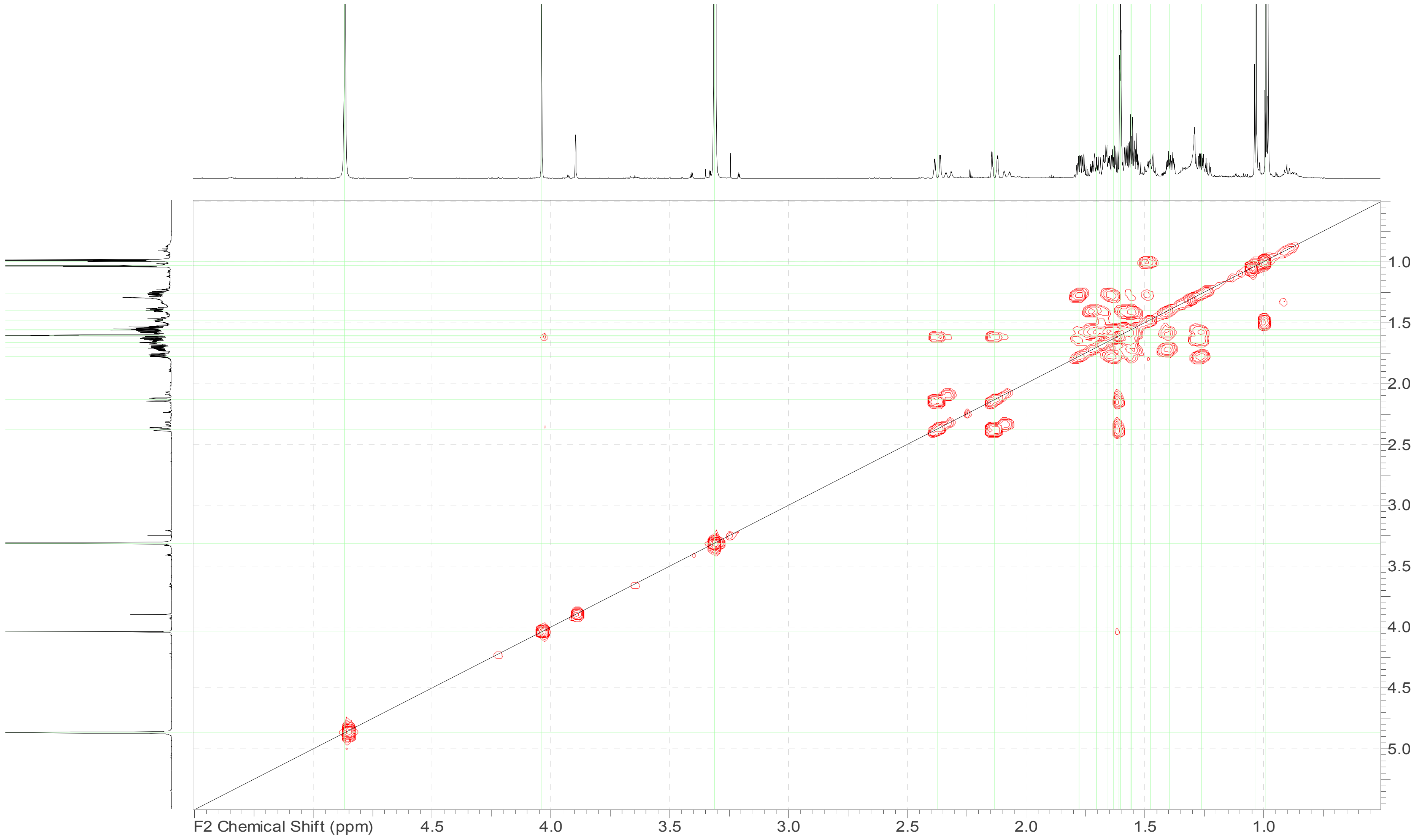

COSY NMR spectrum (500 MHz, CH_3OH-d_4) of **1**.

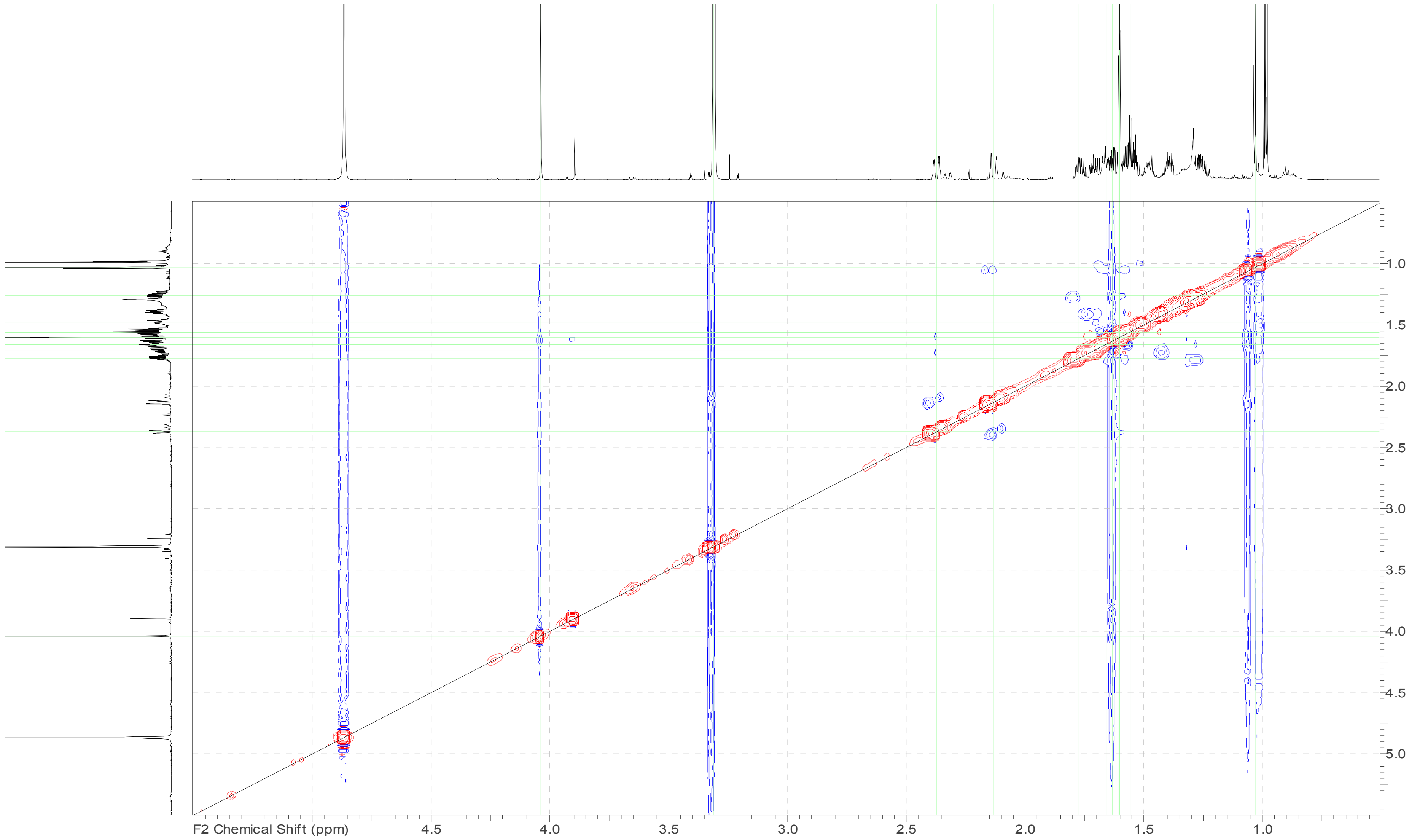

ROESY NMR spectrum (500 MHz, CH_3OH-d_4) of **1**.

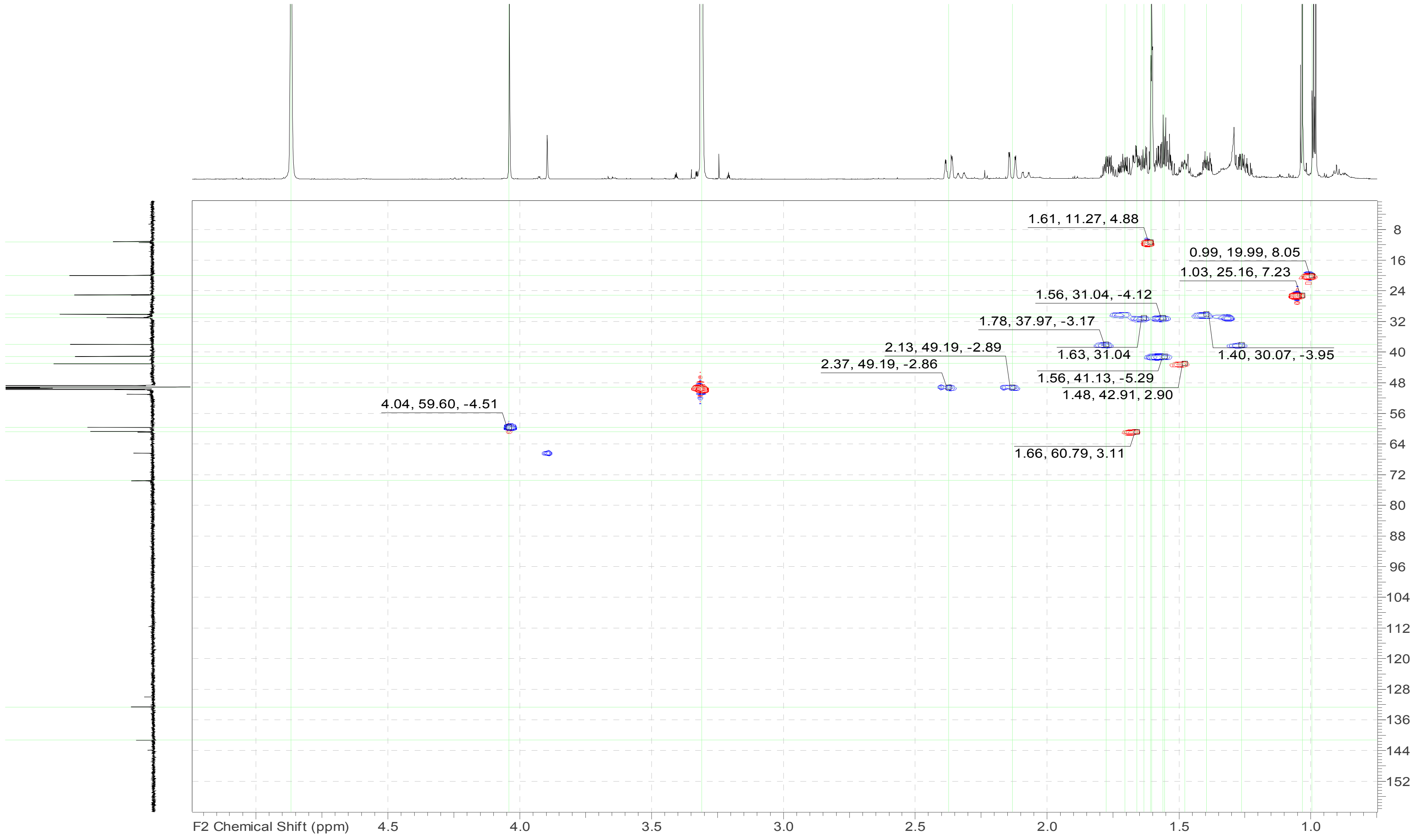

HSQC NMR spectrum (500 MHz, CH_3OH-d_4) of **1**.

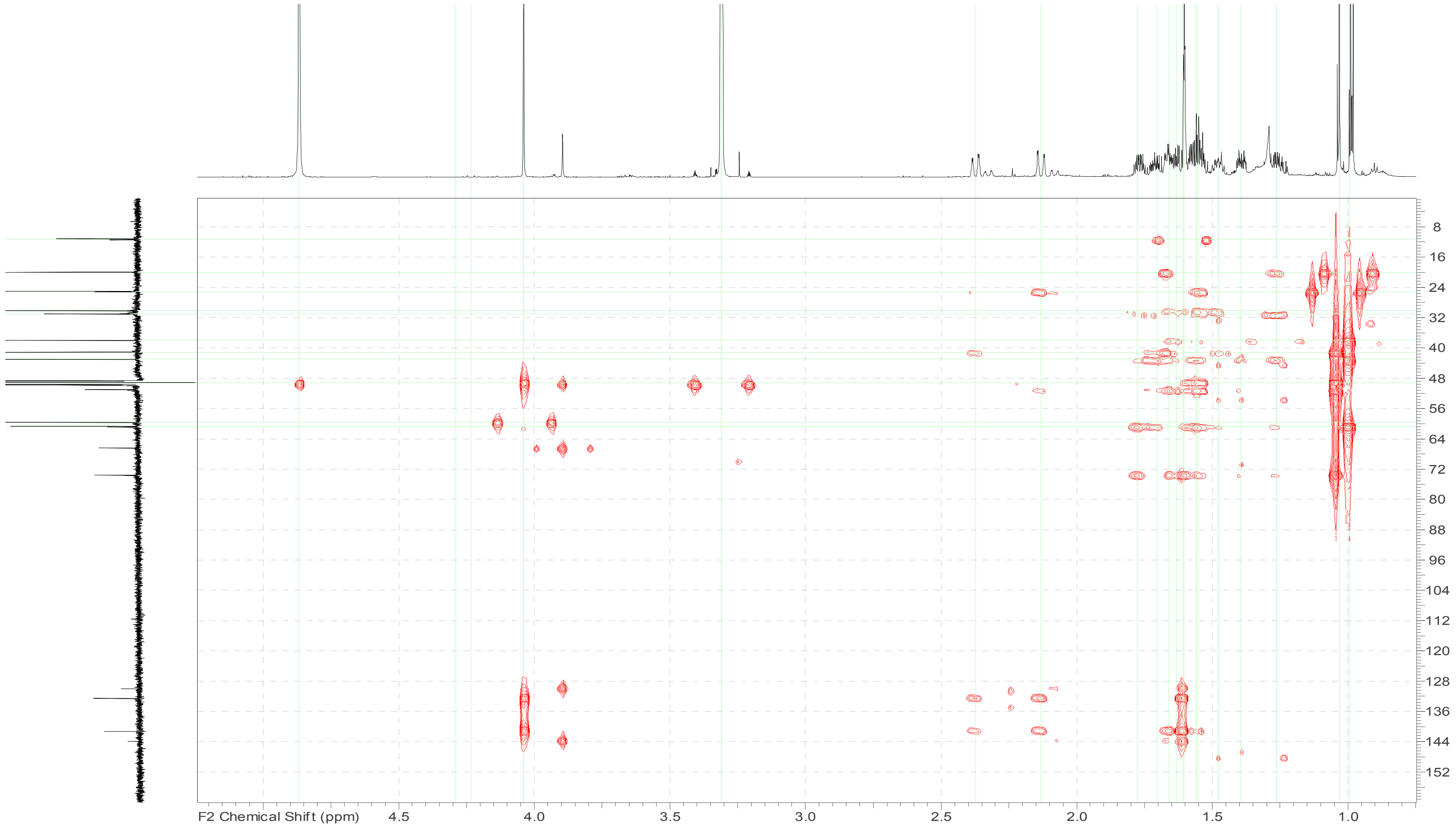

HMBC NMR spectrum (500 MHz, CH_3OH-d_4) of **1**.

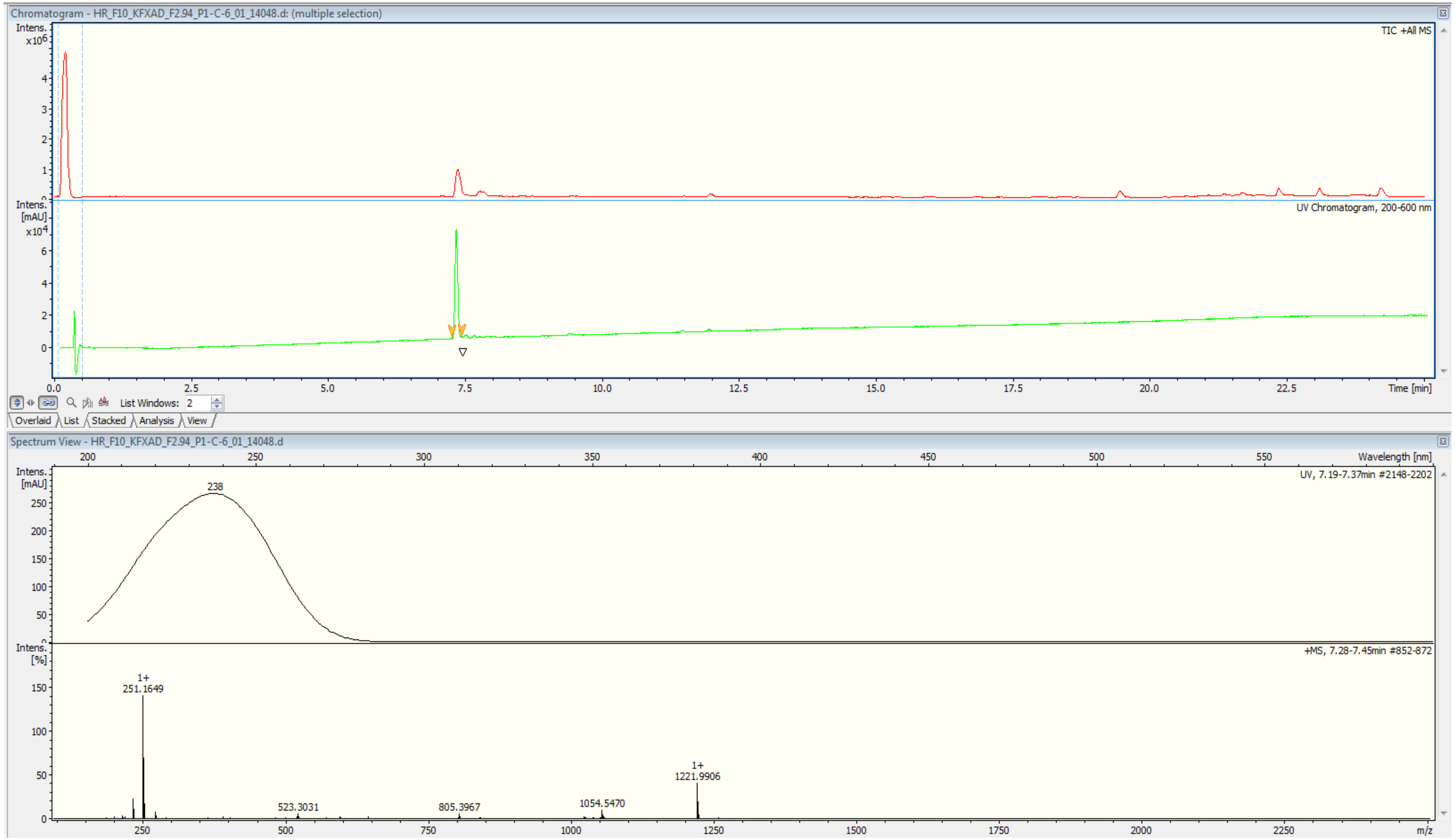

HPLC-HRESIMS data of **2**.

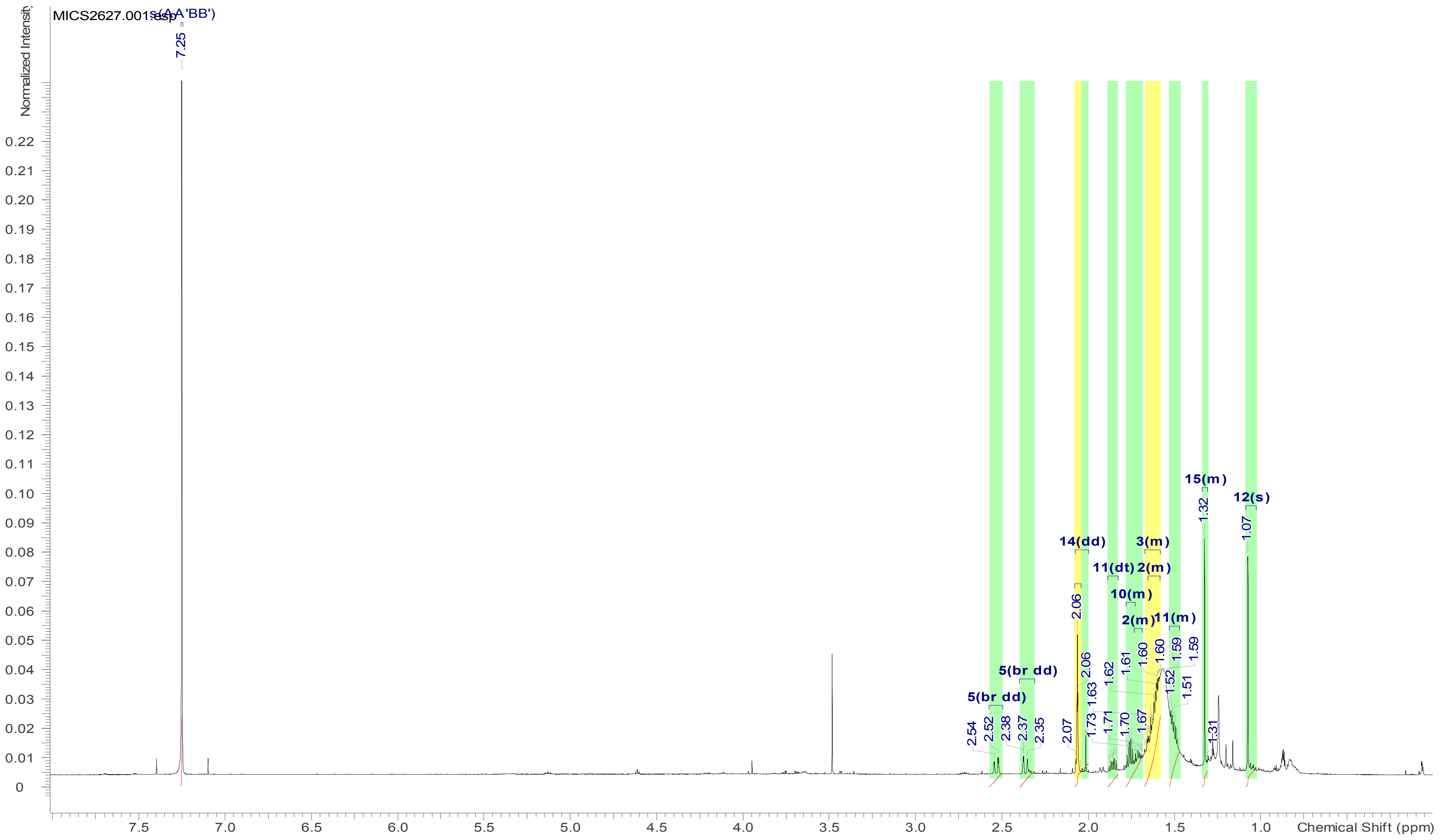

^{1}H NMR spectrum (700 MHz, $CHCl_3$-*d*) of **2**.

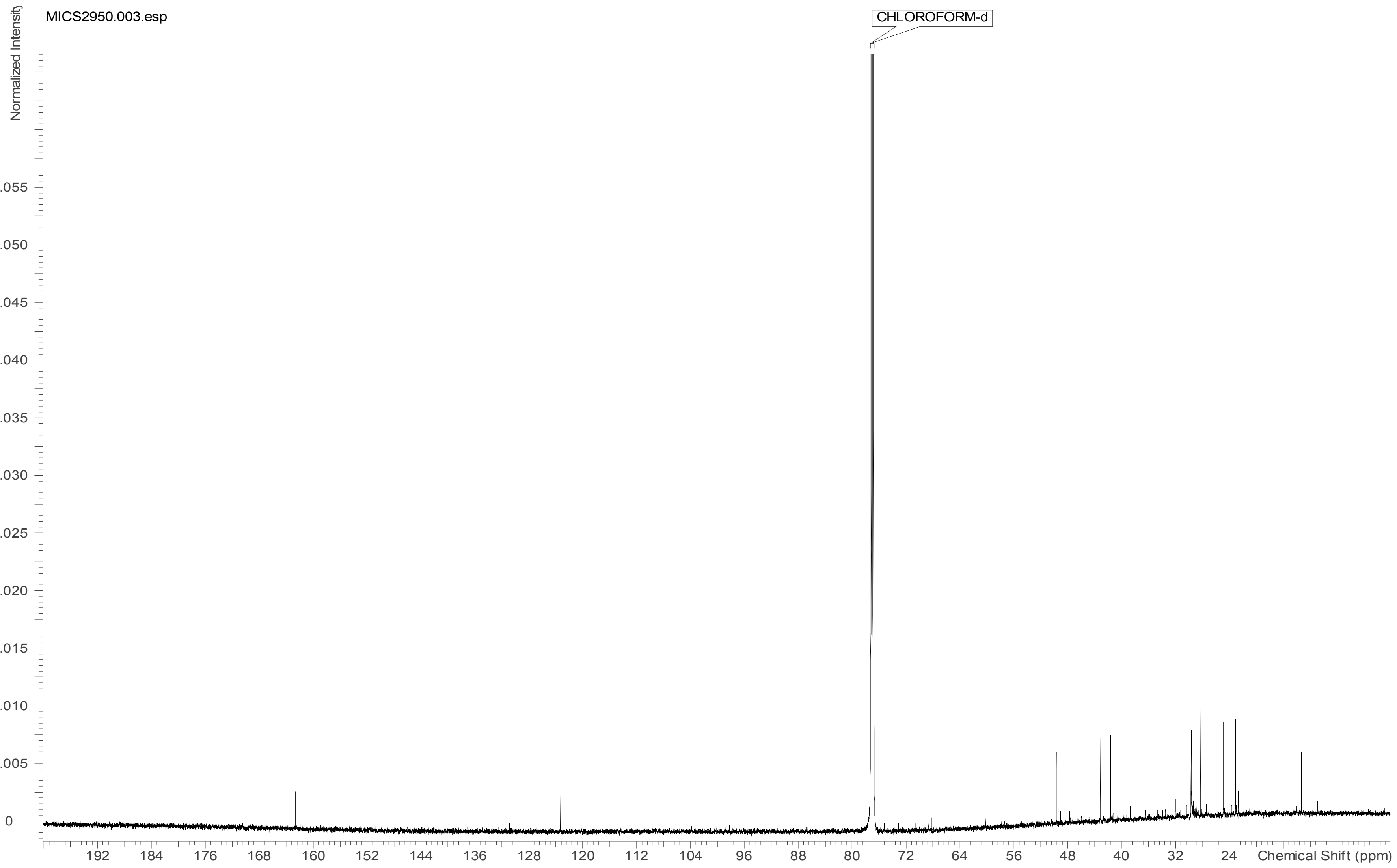

^{13}C NMR spectrum (175 MHz, $CHCl_3$-*d*) of **2**.

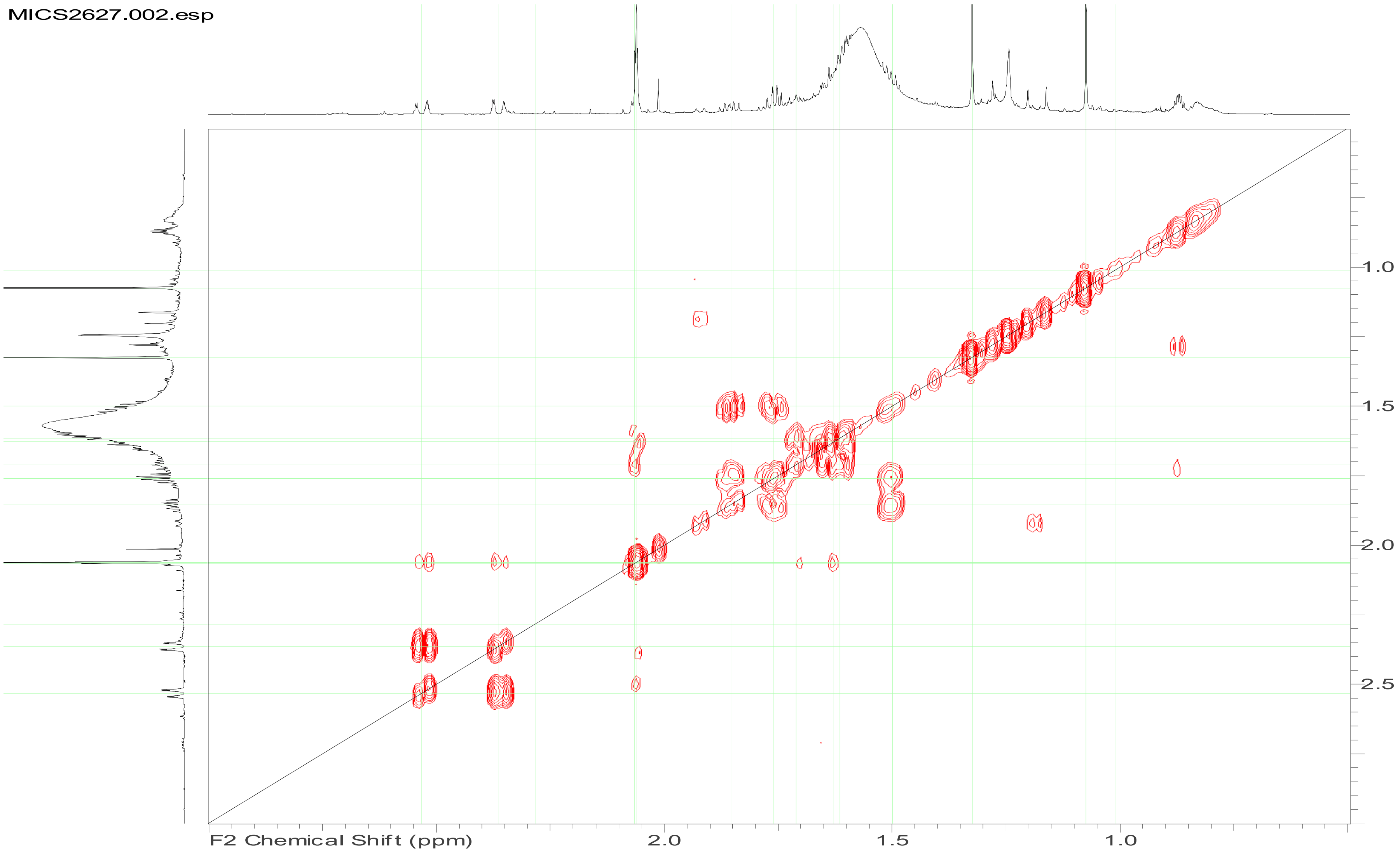

COSY NMR spectrum (700 MHz, $CHCl_3$-*d*) of **2**.

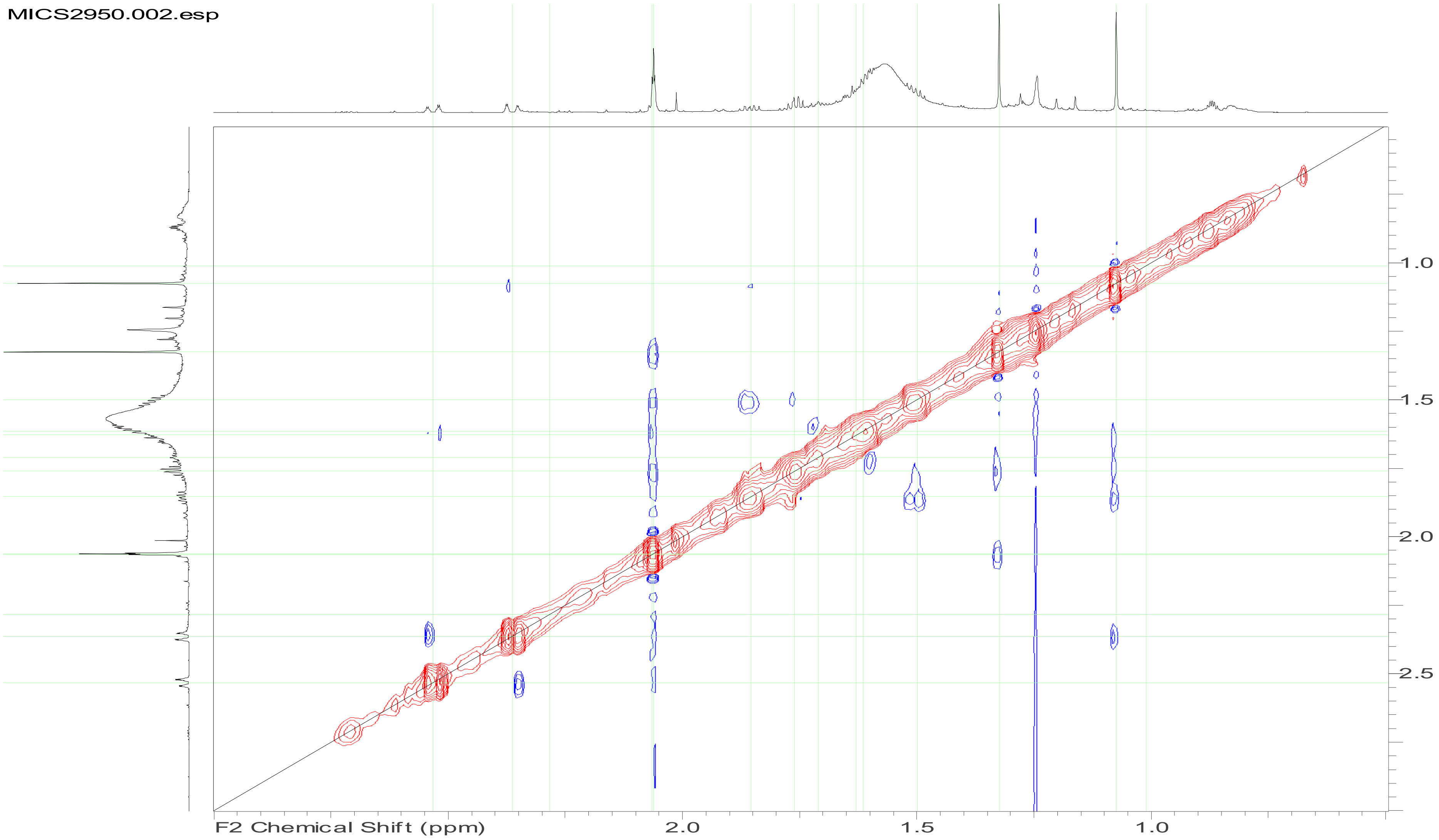

ROESY NMR spectrum (700 MHz, $CHCl_3$-*d*) of **2**.

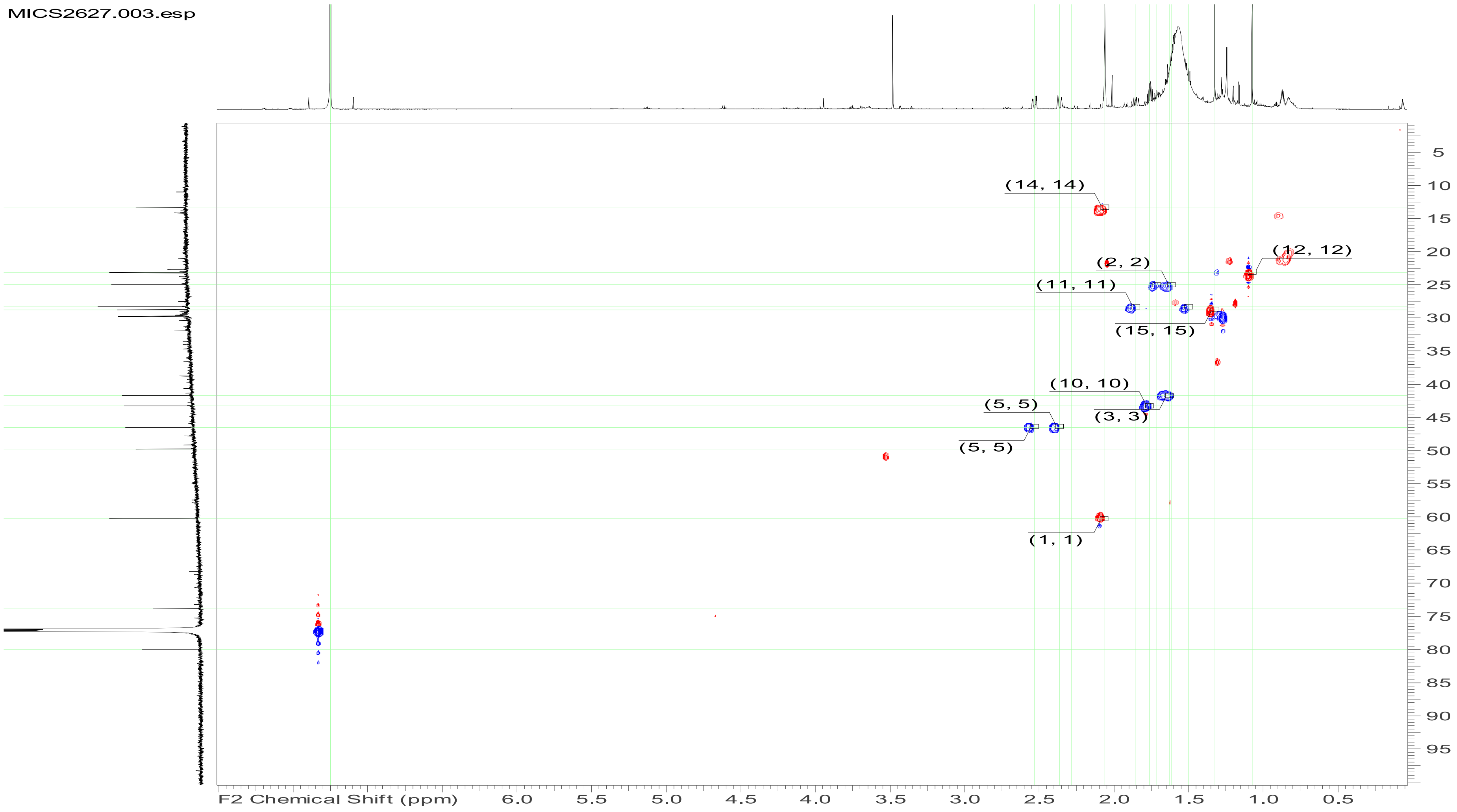

HSQC NMR spectrum (700 MHz, $CHCl_3$-*d*) of **2**.

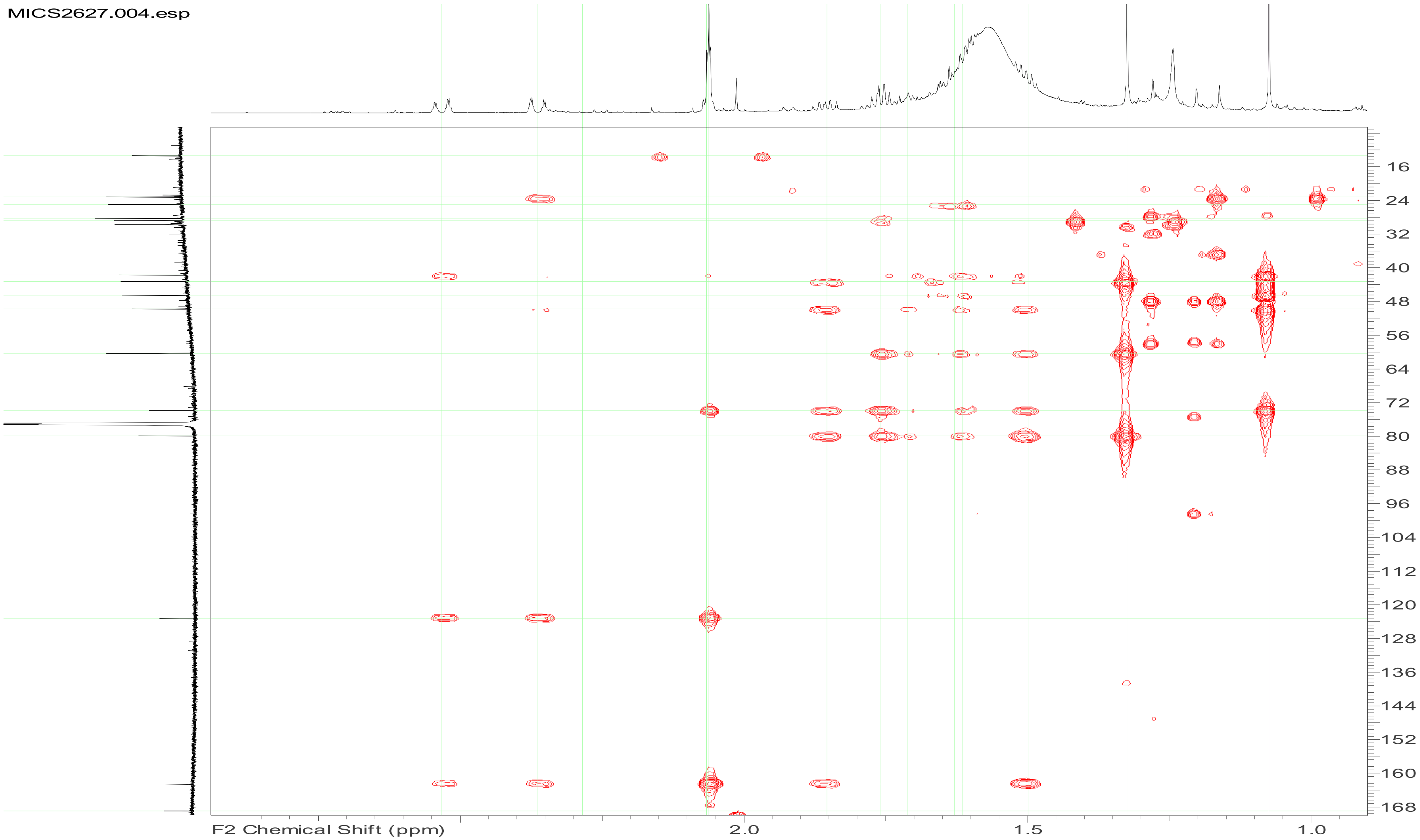

HMBC NMR spectrum (700 MHz, $CHCl_3$-*d*) of **2**.

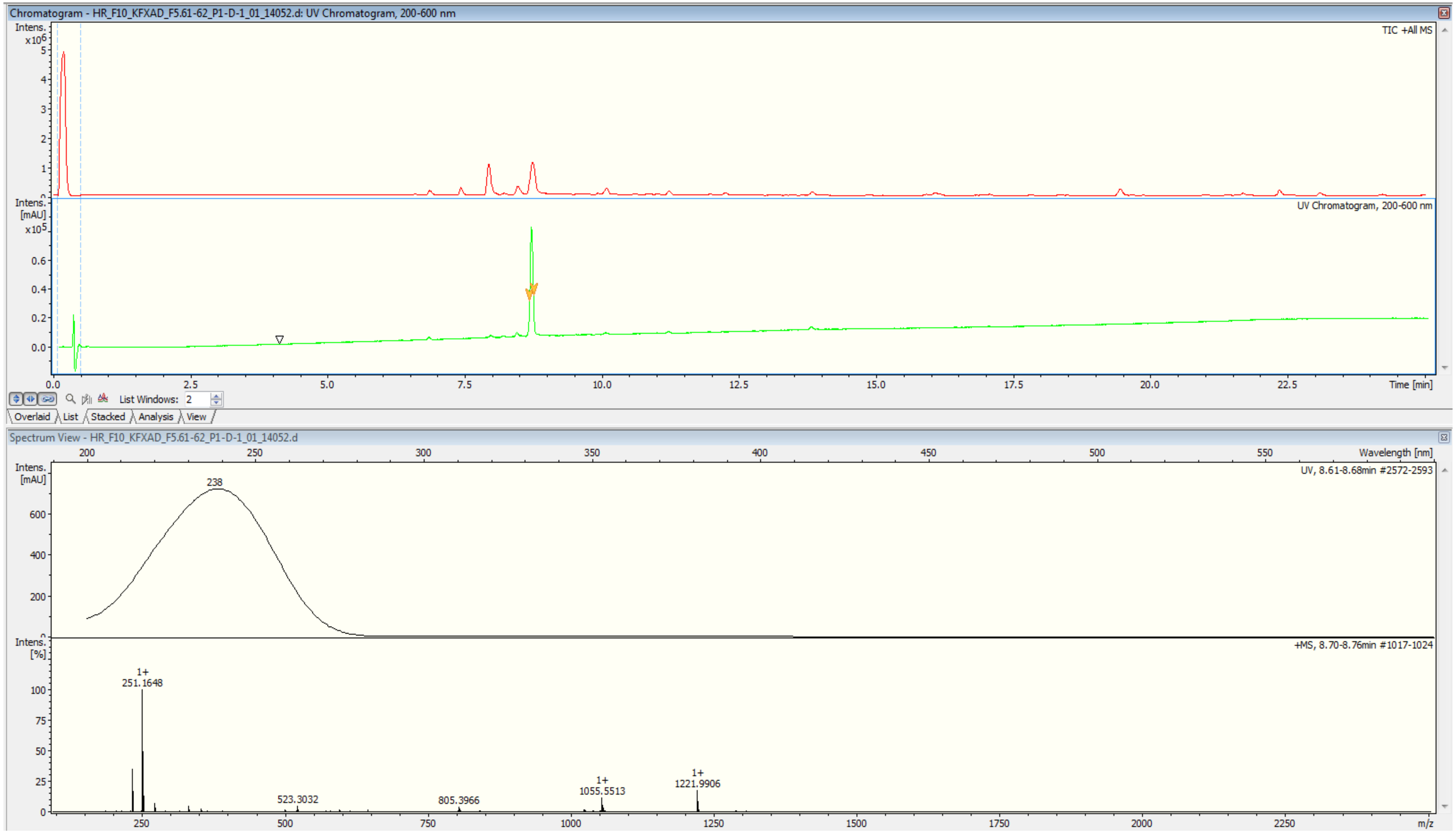

HPLC-HRESIMS data of **3**.

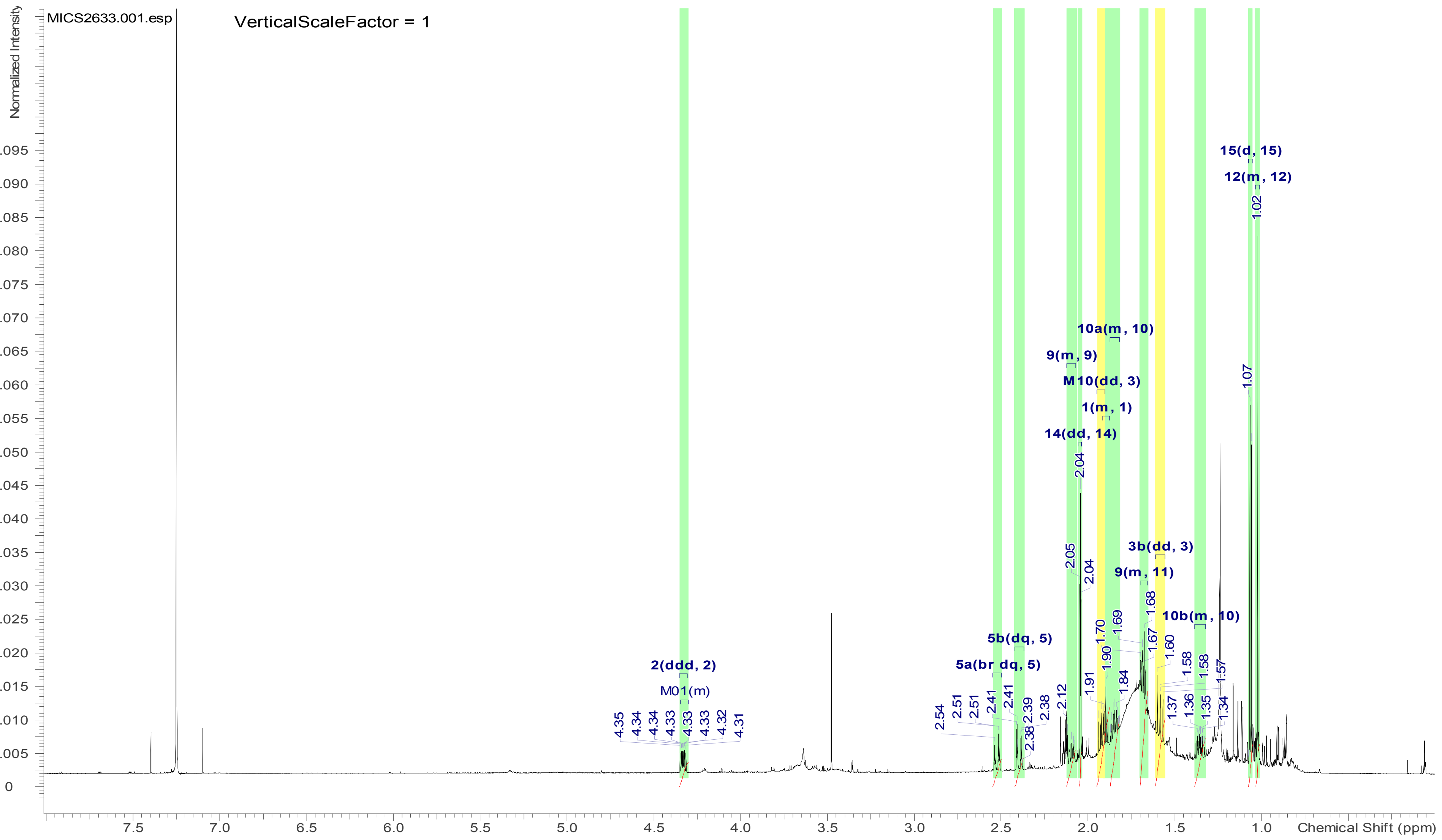

^{1}H NMR spectrum (700 MHz, $CHCl_3$-*d*) of **3**.

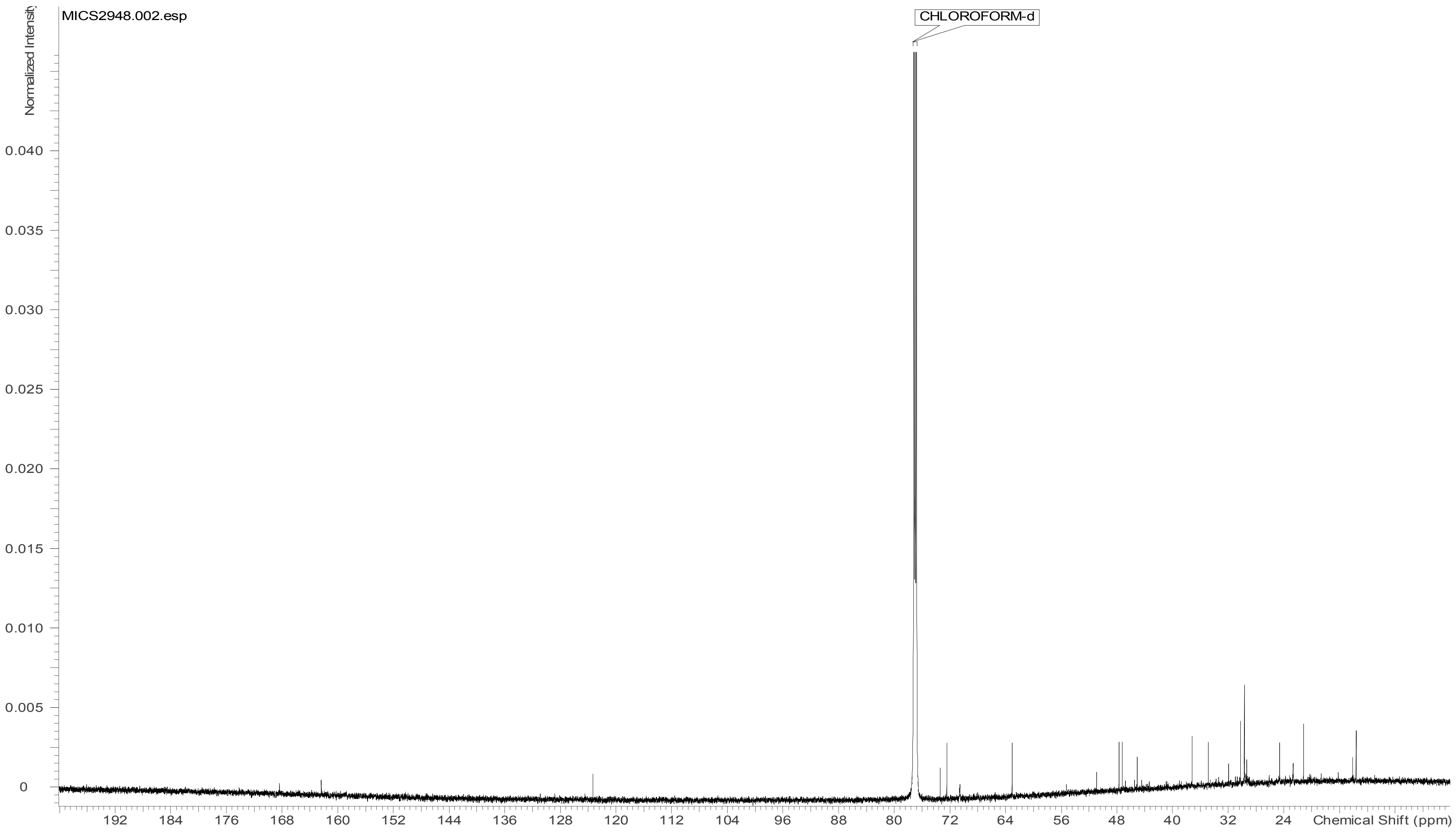

^{13}C NMR spectrum (175 MHz, $CHCl_3$-*d*) of **3**.

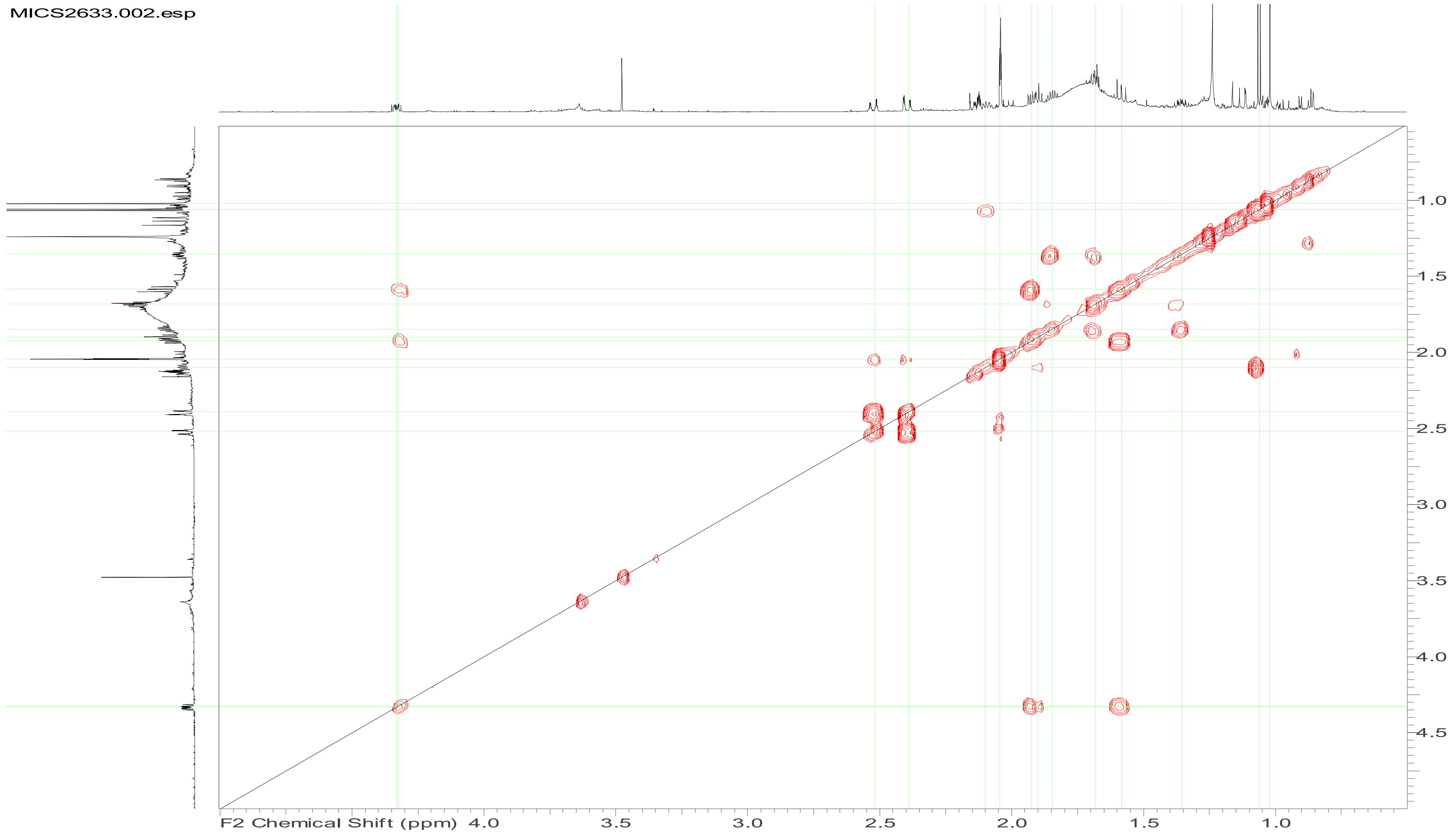

COSY NMR spectrum (700 MHz, $CHCl_3$-*d*) of **3**.

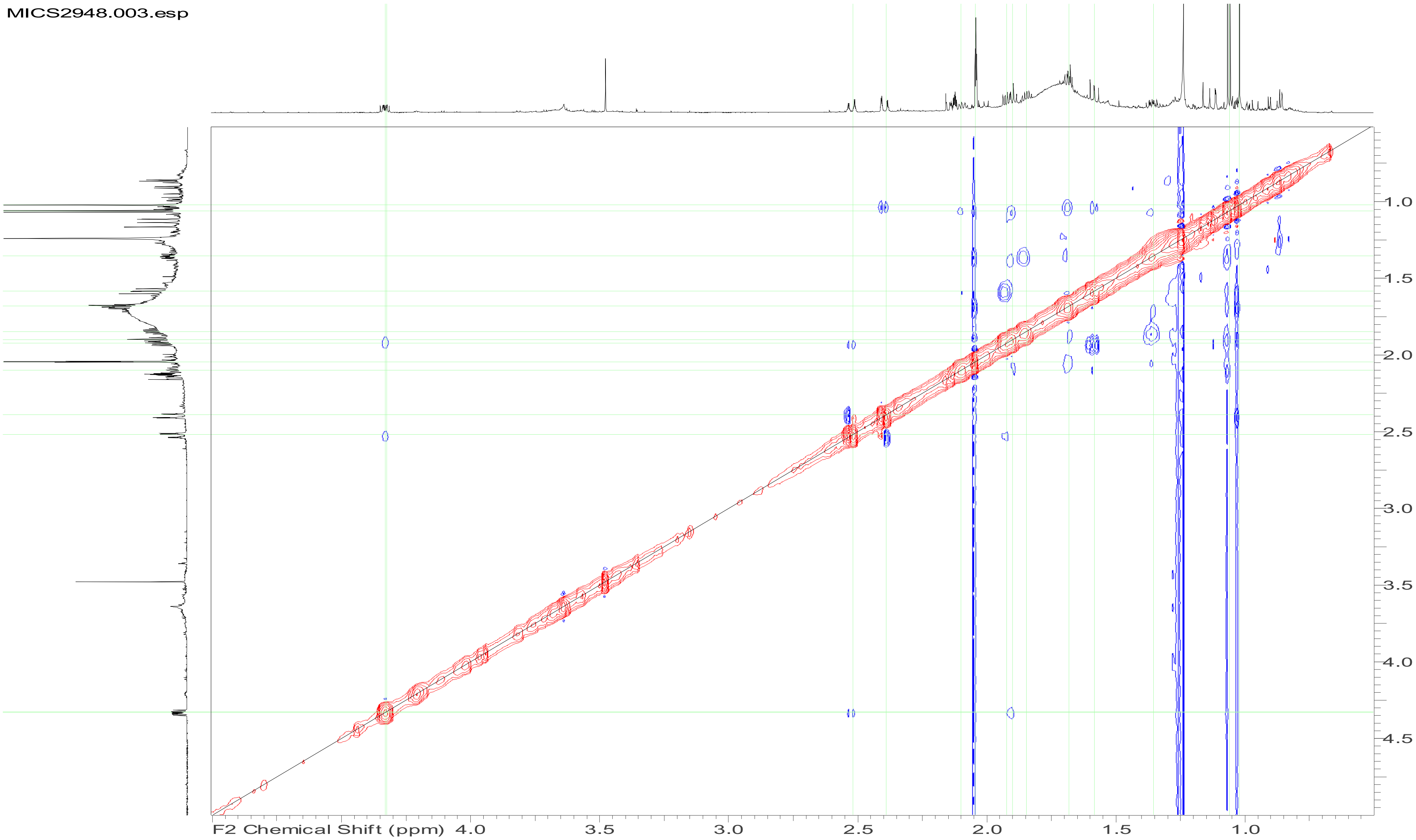

ROESY NMR spectrum (700 MHz, $CHCl_3$-*d*) of **3**.

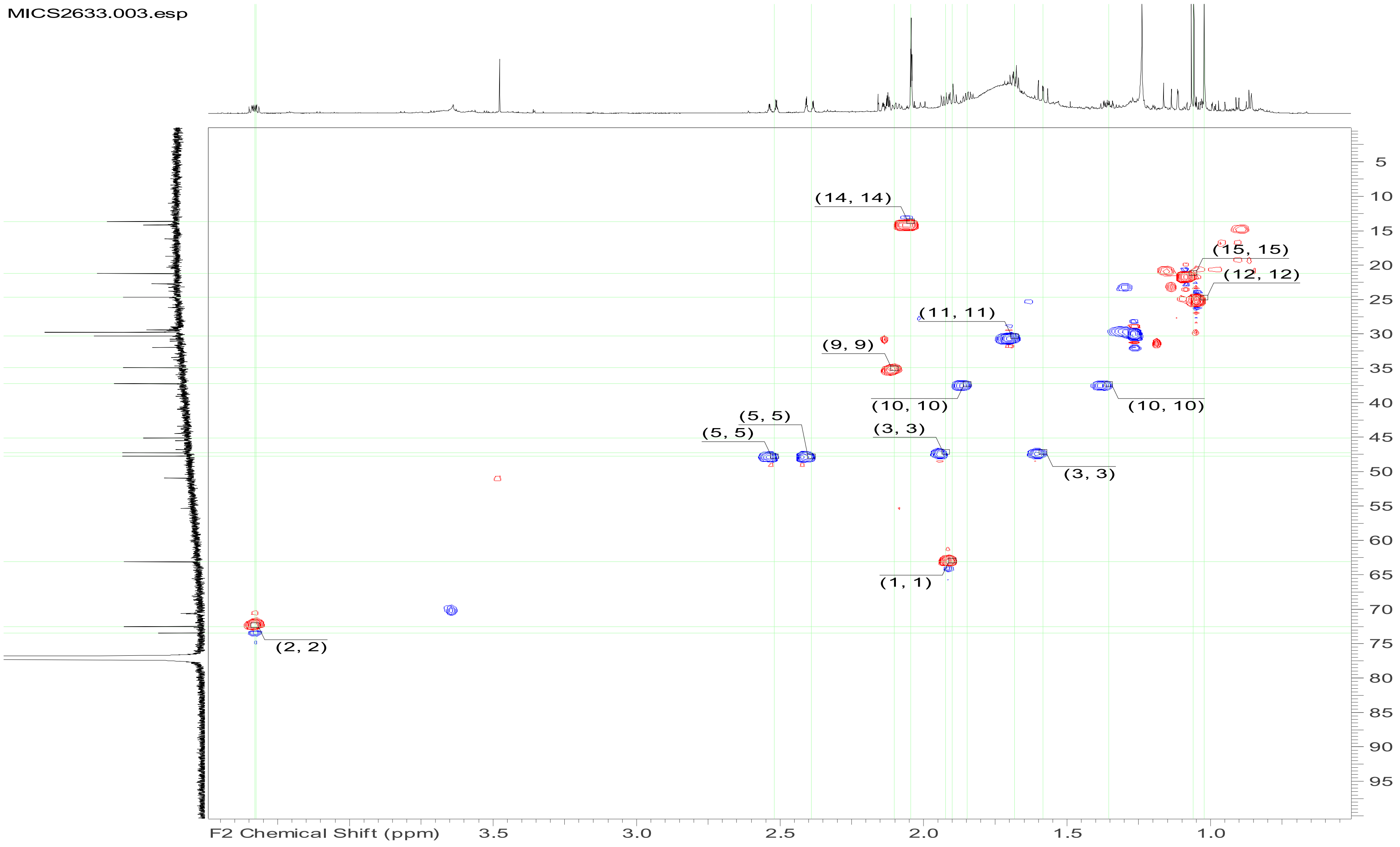

HSQC NMR spectrum (700 MHz, $CHCl_3$-*d*) of **3**.

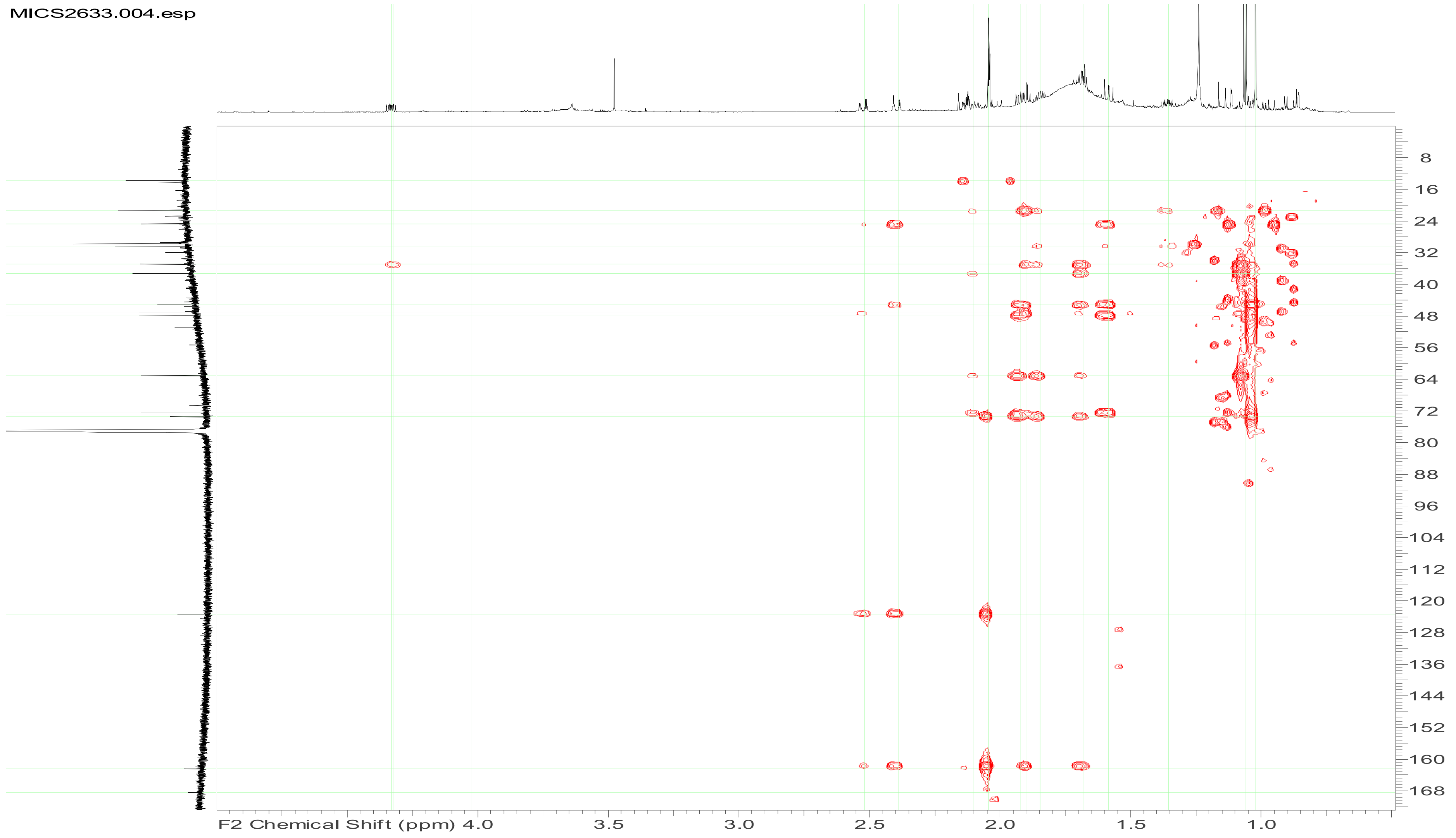

HMBC NMR spectrum (700 MHz, $CHCl_3$-*d*) of **3**.

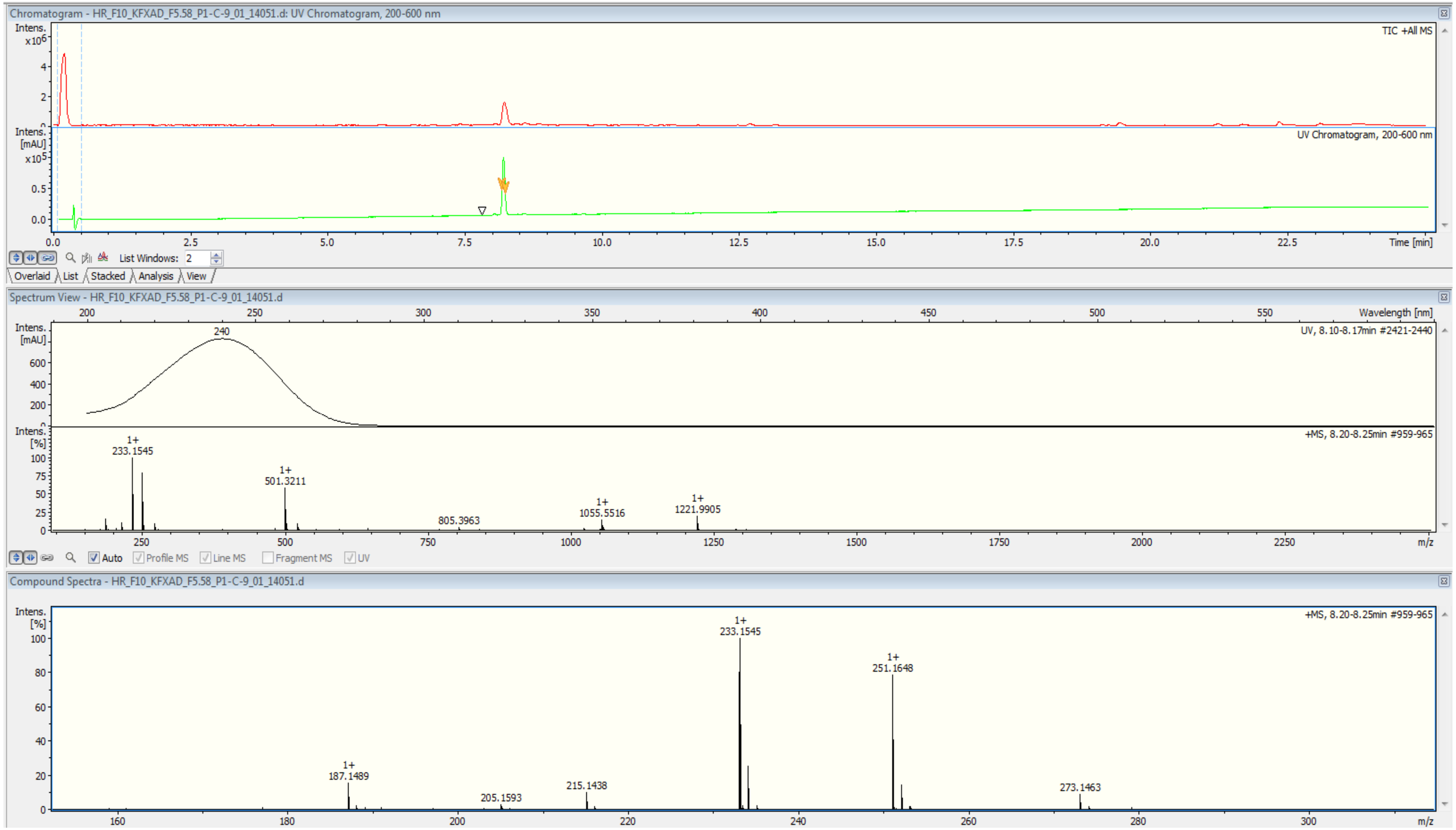

HPLC-HRESIMS data of **4**.

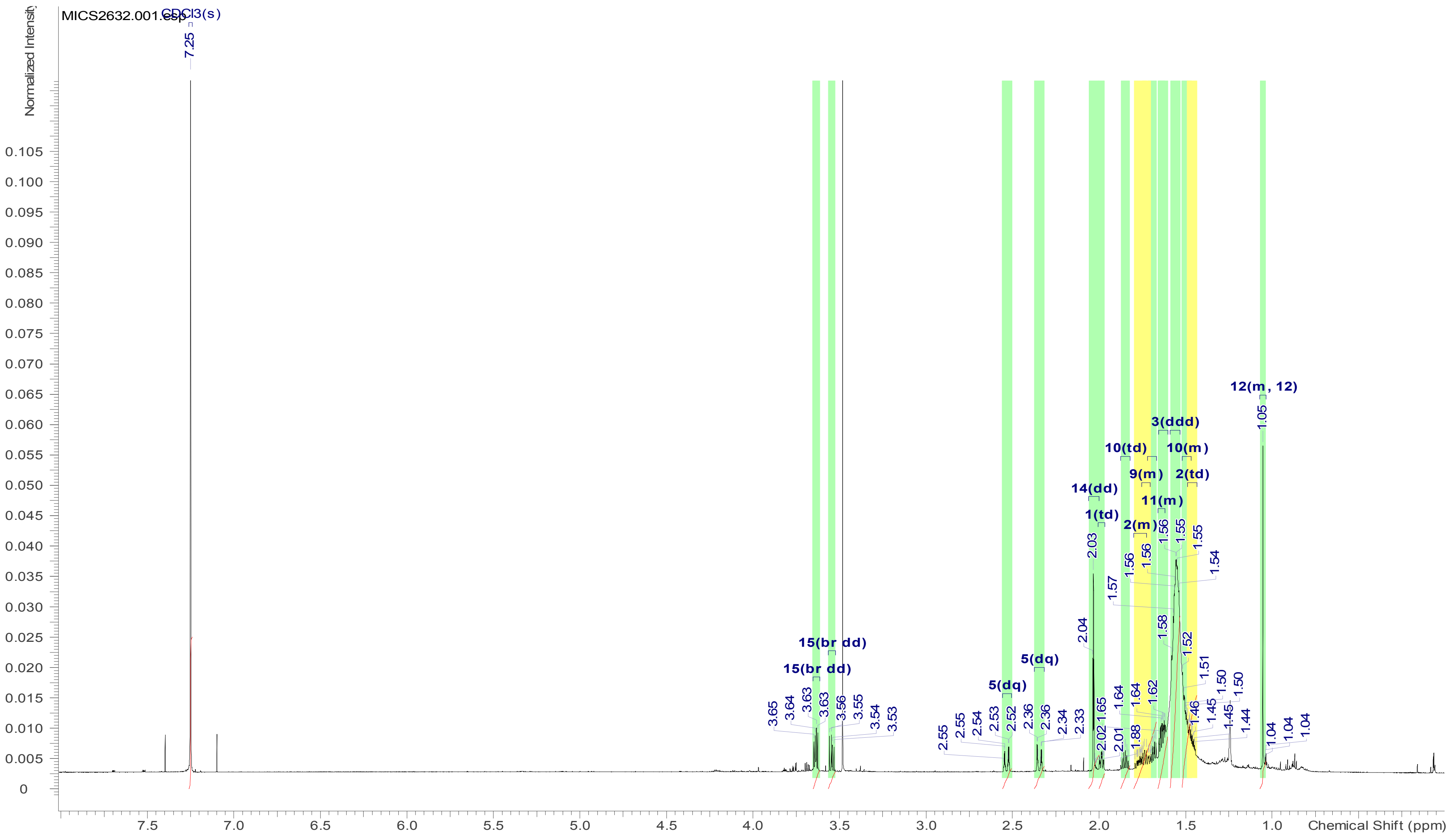

^{1}H NMR spectrum (700 MHz, $CHCl_3$-*d*) of **4**.

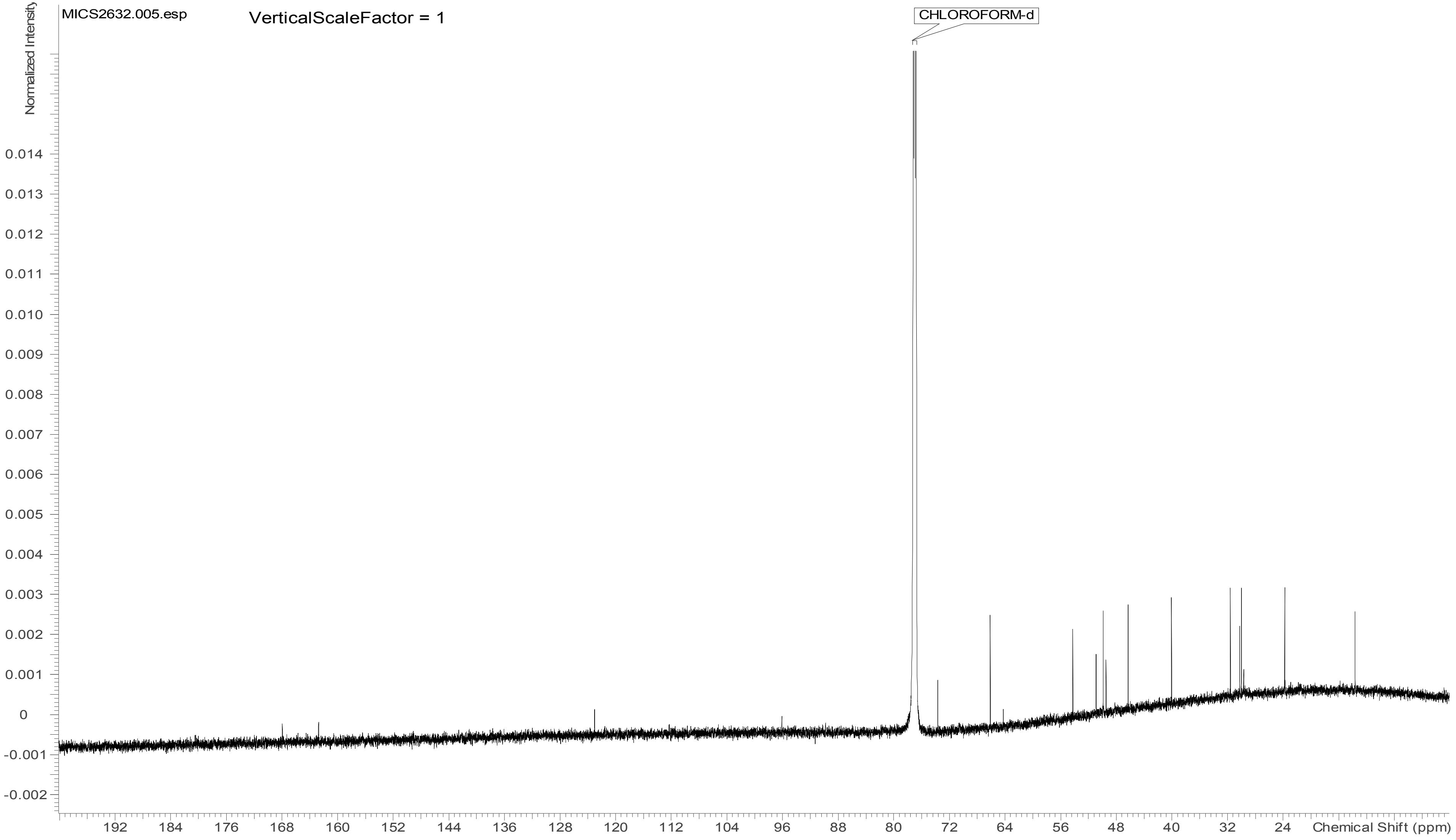

^{13}C NMR spectrum (175 MHz, $CHCl_3$-*d*) of **4**.

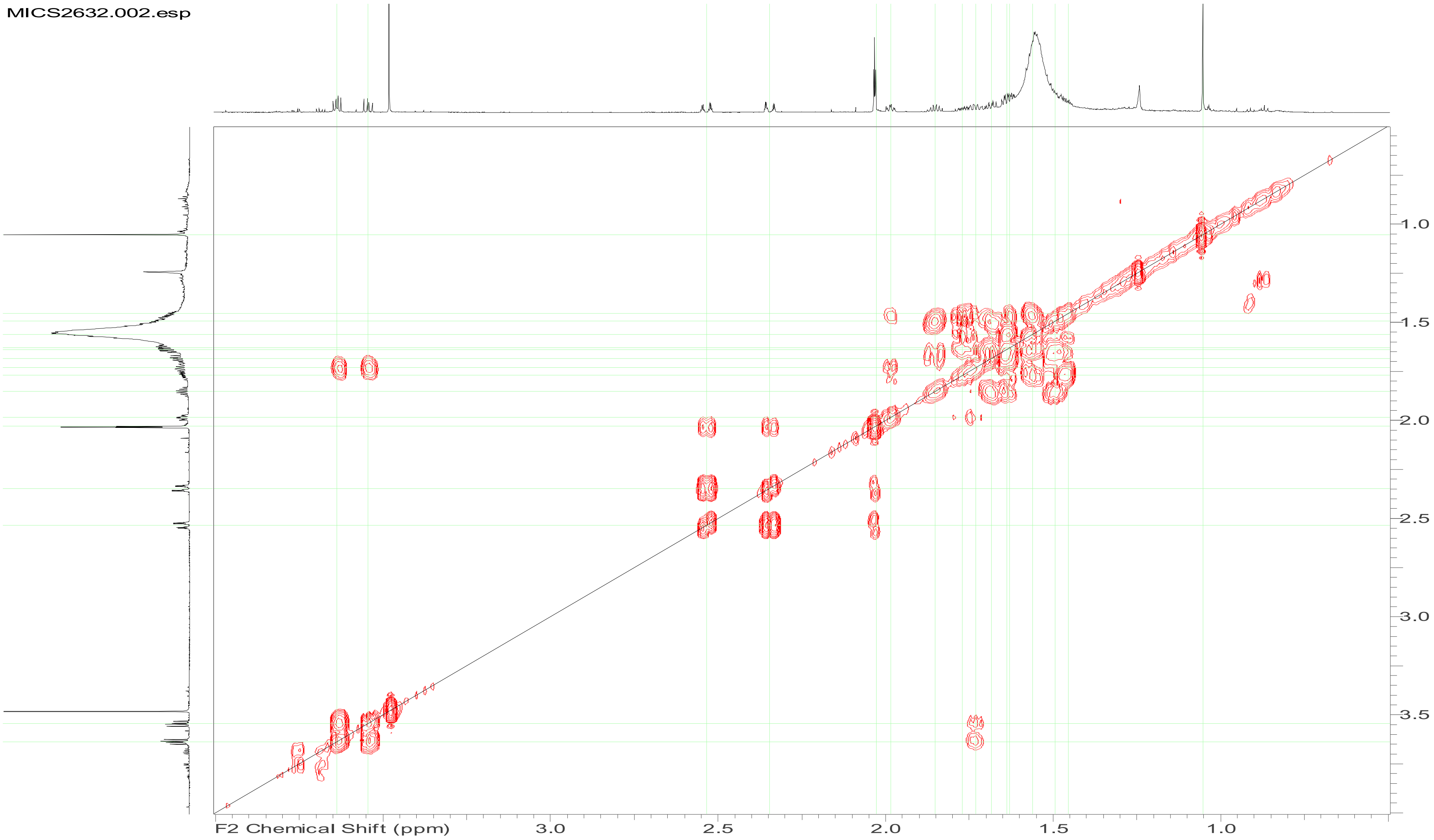

COSY NMR spectrum (700 MHz, $CHCl_3$-*d*) of **4**.

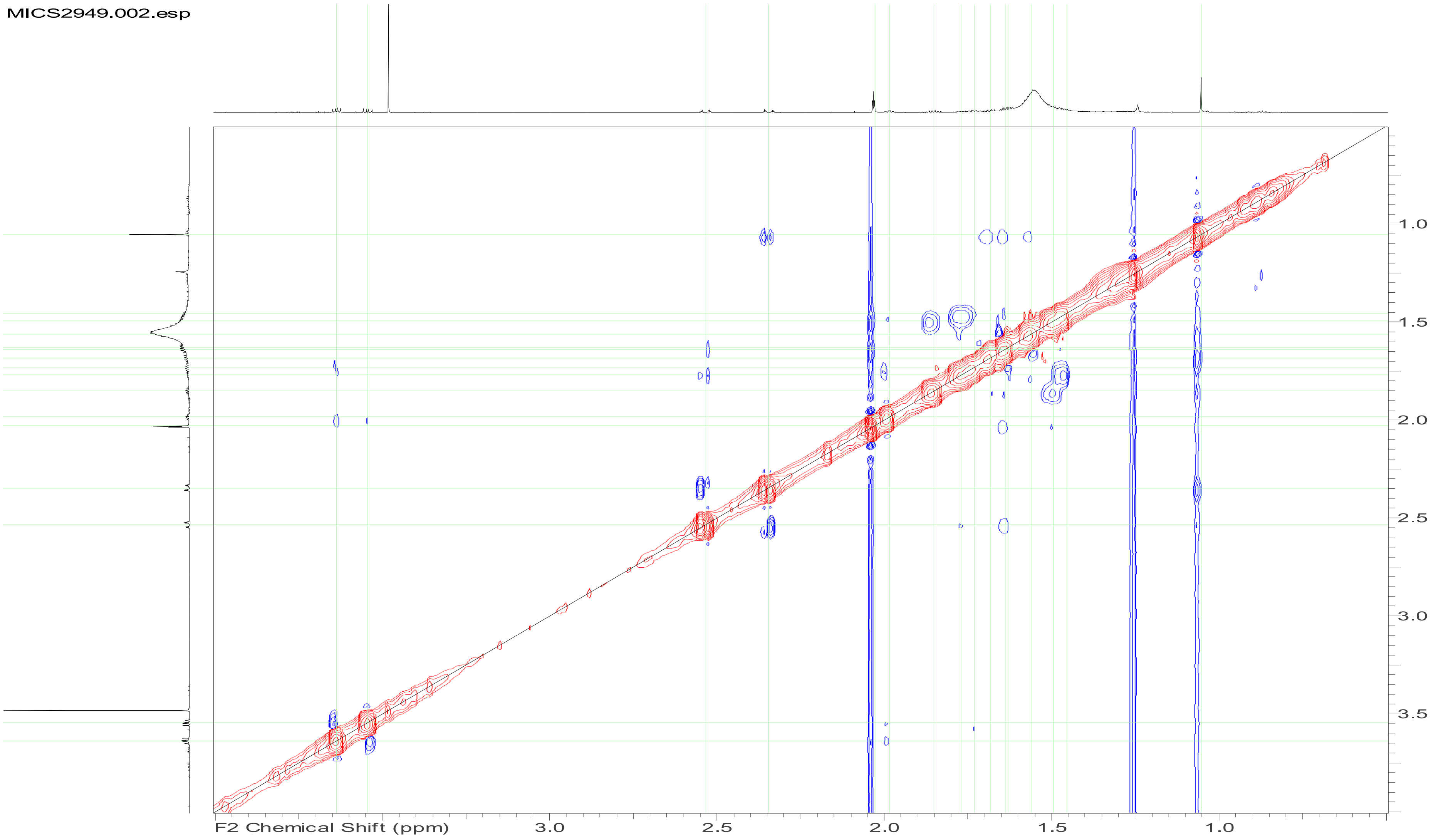

ROESY NMR spectrum (700 MHz, $CHCl_3$-*d*) of **4**.

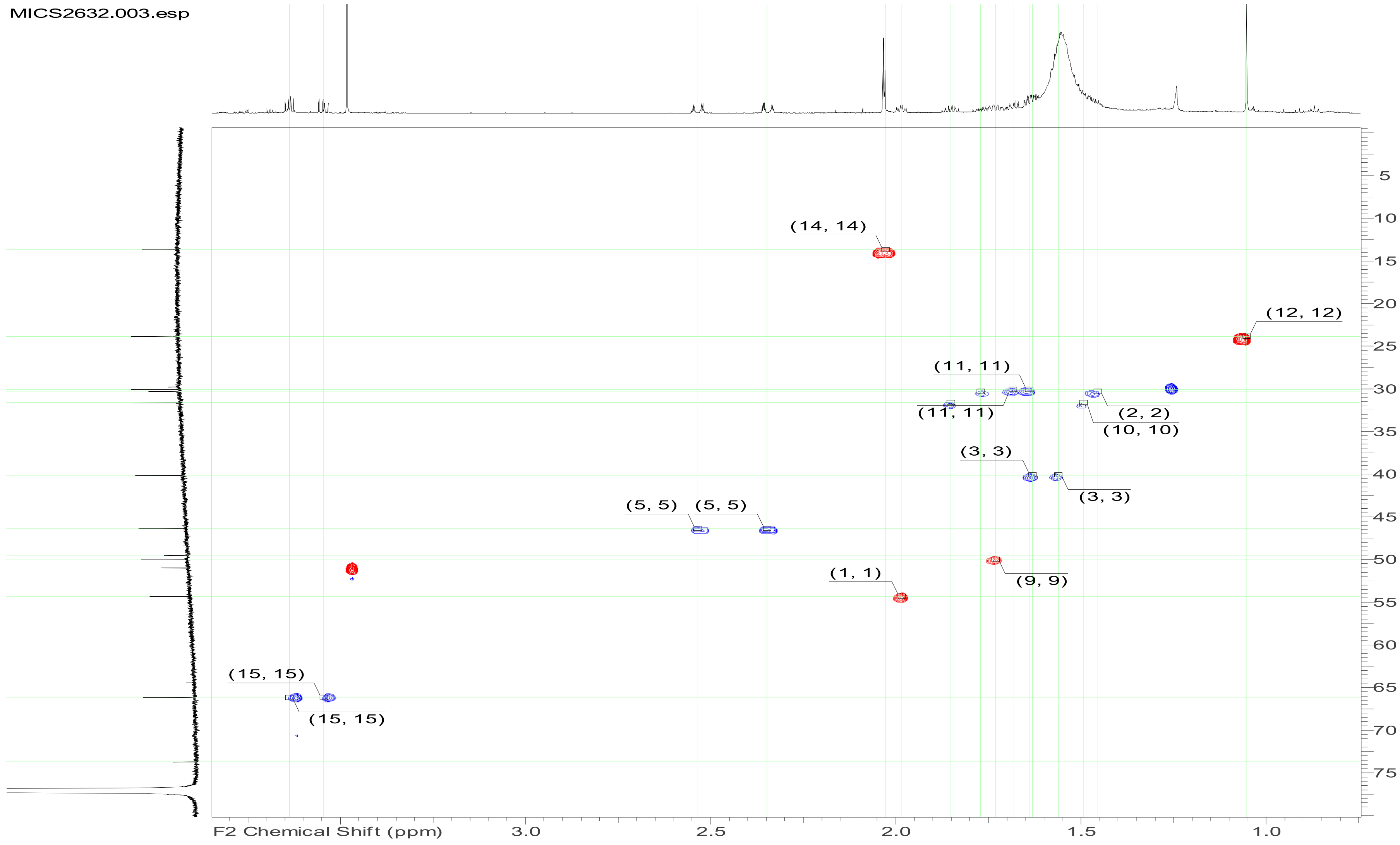

HSQC NMR spectrum (700 MHz, $CHCl_3$-*d*) of **4**.

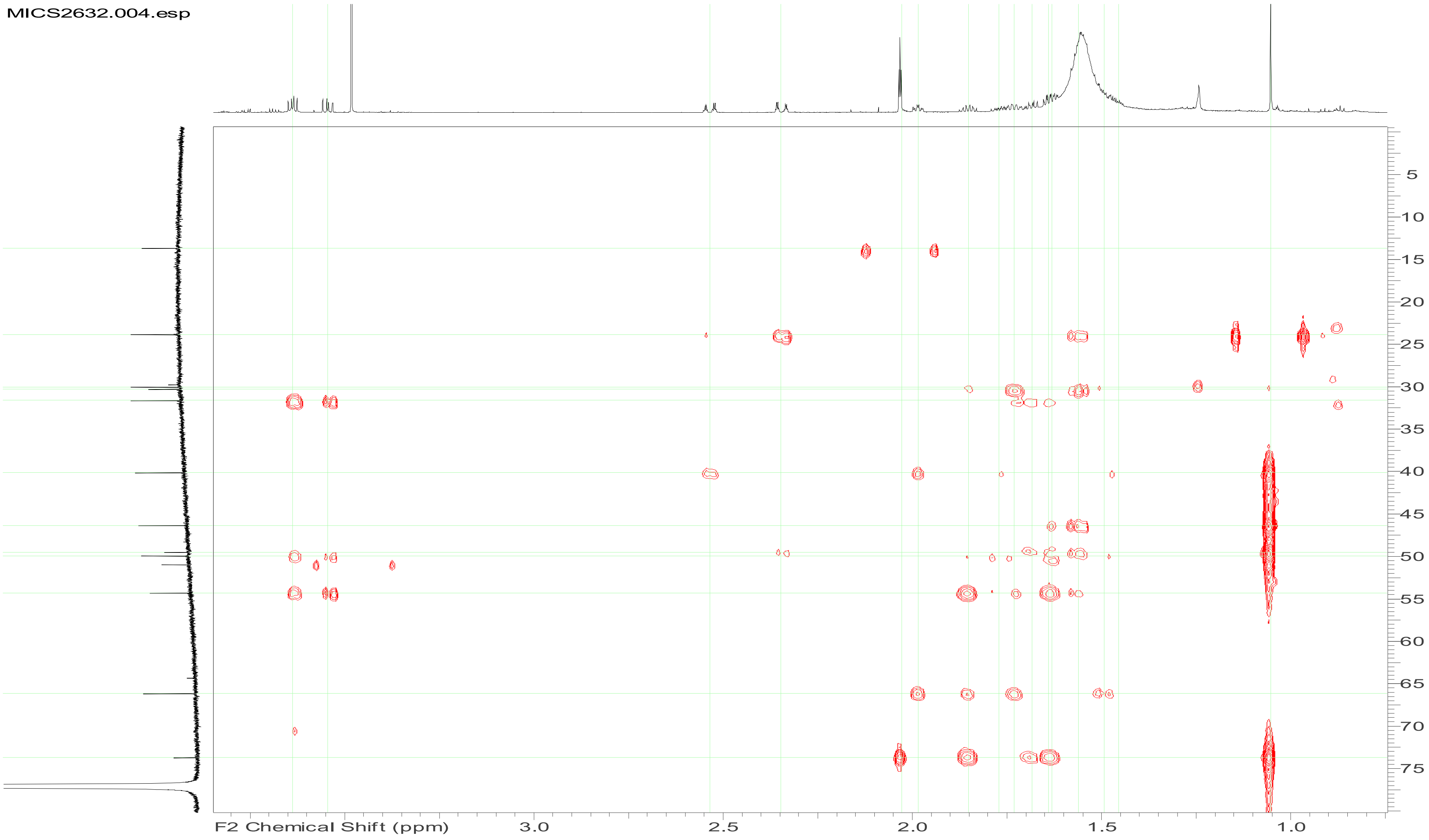

HMBC NMR spectrum (700 MHz, $CHCl_3$-*d*) of **4**.
